# Clonal barcoding with qPCR detection enables live cell functional analyses for cancer research

**DOI:** 10.1038/s41467-022-31536-5

**Published:** 2022-07-04

**Authors:** Qiuchen Guo, Milos Spasic, Adam G. Maynard, Gregory J. Goreczny, Amanuel Bizuayehu, Jessica F. Olive, Peter van Galen, Sandra S. McAllister

**Affiliations:** 1 Division of Hematology, Department of Medicine, Brigham and Women’s Hospital, Boston, MA 02115 USA; 2 Department of Medicine, Harvard Medical School, Boston, MA 02115 USA; 3 Broad Institute of Harvard and MIT, Cambridge, MA 02142 USA; 4 Harvard Stem Cell Institute, Cambridge, MA 02138 USA

**Keywords:** Cell biology, Tumour heterogeneity, Breast cancer, Lineage tracking

## Abstract

Single-cell analysis methods are valuable tools; however, current approaches do not easily enable live cell retrieval. That is a particular issue when further study of cells that were eliminated during experimentation could provide critical information. We report a clonal molecular barcoding method, called SunCatcher, that enables longitudinal tracking and live cell functional analysis. From complex cell populations, we generate single cell-derived clonal populations, infect each with a unique molecular barcode, and retain stocks of individual barcoded clones (BCs). We develop quantitative PCR-based and next-generation sequencing methods that we employ to identify and quantify BCs in vitro and in vivo. We apply SunCatcher to various breast cancer cell lines and combine respective BCs to create versions of the original cell lines. While the heterogeneous BC pools reproduce their original parental cell line proliferation and tumor progression rates, individual BCs are phenotypically and functionally diverse. Early spontaneous metastases can also be identified and quantified. SunCatcher thus provides a rapid and sensitive approach for studying live single-cell clones and clonal evolution, and performing functional analyses.

## Abstract (web summary)

DNA barcoding methods for the analysis of clonal heterogeneity in cancer have been limited in terms of throughput and practical requirements. Here, the authors develop SunCatcher, a rapid and sensitive barcoding approach for live single-cell clonal evolution analysis, and use this method to study breast cancer cell populations.

## Introduction

Cell fate mapping and lineage tracing techniques have been useful for describing heterogeneity and clonal dynamics of complex tissues in both normal and pathological settings. Early insights into hematopoiesis were derived from analyzing karyotypes, assessing heterozygosity of particular genes, and analyzing viral integration patterns^1–3^. Although limited by their lack of sensitivity and qualitative nature, those techniques enabled the first effective tracking of clonal populations.

Understanding cellular heterogeneity and clonal fitness is particularly important to cancer research. Clinically, intratumoral diversity inversely correlates with therapeutic response, metastatic ability, and patient survival^4–6^. Although differences in the mutational profile of tumor cell subpopulations may be the best-documented parameter of tumor heterogeneity, it is ultimately the functional heterogeneity, a result of both genetic and non-genetic sources of heterogeneity, that impacts the course of disease progression^7–11^. Large-scale sequencing efforts and technologies such as single-cell RNA sequencing and single-nucleus sequencing have revealed the diverse mutational and transcriptional landscape of human cancers^12–16^. Nevertheless, we still have much to learn about the effects of functional heterogeneity on disease progression.

Accounting for and properly modeling tumor heterogeneity and cell plasticity in experimental and pre-clinical settings is thus crucial for improving cancer treatment outcomes. For example, we previously reported that functional heterogeneity can impact data interpretation in studies that employ gene editing and designed a novel CRISPR/Cas9 protocol that incorporates a single-cell cloning step prior to gene editing in order to generate appropriately matched control and edited cells^17^. Clonal dynamics – driven by clone frequency, spatial proximity, heterotypic interactions, and/or cell plasticity – can profoundly affect disease progression^4,10,18,19^.

The advent of molecular barcoding techniques, in which short, non-coding DNA sequences are stably integrated into the genomes of complex cell populations using viral infection, enables simultaneous tracking of thousands of clonal populations^20–22^. In cancer research, DNA barcoding techniques have been used to successfully trace clonal dynamics in heterogeneous tumor cell populations^23–30^. Those early tracing techniques utilized barcode library infection methods in which 10^4^–10^7^ barcodes are simultaneously introduced into a single population of tumor cells. While such high-complexity barcode libraries enable longitudinal quantification of clonal diversity in various experimental settings, they do not enable characterization, functional analyses, or manipulation of the cellular populations of interest. More recently, several elegant and sophisticated methods have been developed to trace as well as study certain select barcoded cells, including expressed barcodes and CRISPR-based clone retrieval^11,31–39^. Nevertheless, some of those methods are limited in the numbers of barcoded cells that can be selected for study, particularly for the negatively selected clones, and the techniques are not always widely accessible.

Here, we report a molecular barcoding approach, termed SunCatcher, that provides a rapid, inexpensive, and highly sensitive method to detect, identify, quantify, isolate, and study individual clones, or mixtures of clones, of interest. SunCatcher is based on generating single-cell-derived clonal populations from any complex cell population and infecting each clonal population with a unique, heritable molecular barcode. The barcoded clones can be studied individually or combined to generate custom pools of clones. As such, SunCatcher enables both longitudinal and retrospective analysis of the molecular, phenotypic, and functional basis for heterogeneity and clonal fitness during experimentation. Importantly, our approach enables the study of clones that are negatively selected during a given process, allowing their specific vulnerabilities to be uncovered, which might not be possible with conventional molecular barcoding techniques. We apply SunCatcher to barcode individual clones from various mouse and human breast cancer cell lines. Heterogeneous pools of barcoded cells reliably reproduce the original in vitro and in vivo growth and tumor progression rates of the original parental cell lines. The SunCatcher approach is also highly effective for detecting and identifying early spontaneous skeletal and visceral metastases, and cells that underwent immune editing or escape during disease progression. We envision that SunCatcher can be applied to any cell-based studies and believe that it will serve as a useful, widely accessible tool for research and discovery.

## Results

### Development of the SunCatcher clonal barcoding method

**Fig. 1:**
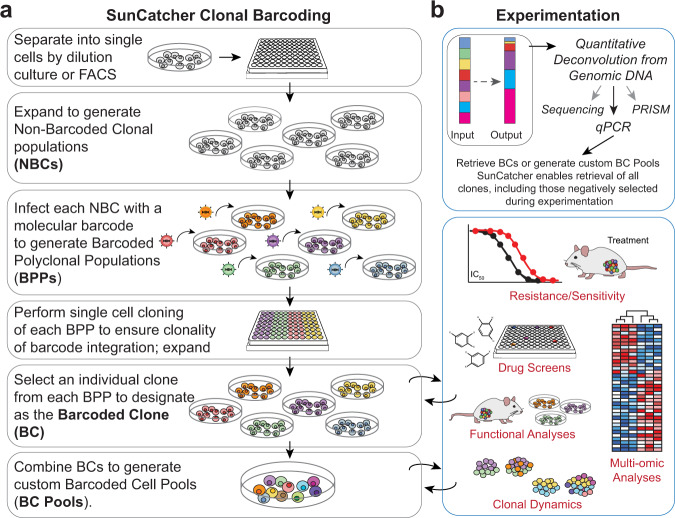
SunCatcher clonal barcoding and functional analyses. **a** SunCatcher utilizes two rounds of single cell cloning to ensure that each subclone has only 1 unique barcode and that each cell within that subclone contains the same barcode insertion site. Due to the single cell cloning approach, custom BC pools of any combination can be designed. (FACS, fluorescence-activated cell sorting; NBC, non-barcoded clone; BPP, barcoded polyclonal population (polyclonal for barcode insertion site); BC, barcoded clone; BC Pool, population containing multiple BCs). **b** BCs or BC Pools (input) can be entered into any experiment and detected at end point (output) using various deconvolution approaches. Deconvolution by quantitative polymerase chain reaction (qPCR) provides a cost-effective, rapid, and highly sensitive method for detecting and quantifying BCs. SunCatcher enables retrieval of all clones, including those negatively selected during experimentation, for further analyses and/or design and testing of custom BC Pools.

We developed a clonal barcoding system, which we call SunCatcher, that enables longitudinal and retrospective analyses of live cells of interest. First, single cells are isolated from a heterogeneous parental population of cells, by either dilution passage or fluorescence-activated cell sorting (FACS) based on cell-surface markers of interest, into individual wells of 96-well plates (Fig. 1a). Each single-cell-derived colony is then expanded, after which aliquots of cells from each clonal population are viably frozen to generate stocks of non-barcoded clones (NBCs). Maintenance of NBC stocks is useful should any challenges arise in subsequent steps of the protocol resulting in loss of a given clonal population, in which case that clone can be recovered.

Each NBC population is then infected with a unique molecular barcode (Fig. 1a) that enables each clonal population to be tracked and quantified. Here, we used the PRISM collection of distinct lentiviral vectors that each contains a unique, heritable 24-base-pair (bp) DNA barcode sequence designed to avoid sequence homology with the genome^29,40^. We refer to these cells as barcoded polyclonal populations (BPPs; Fig. 1a) since the barcodes are randomly inserted into the genome. To ensure the clonality of the barcode insertion site, a subsequent round of single cell cloning of each BPP is performed; those subclones are expanded and aliquots of each are viably frozen (Fig. 1a). Next, one population for each barcode is randomly selected to represent the barcoded clone (BC). At this stage, each BC has a single, unique 24-bp barcode and is clonal for the lentiviral insertion site. Each BC population is aliquoted into cryovials and viably frozen to generate BC stocks. The BCs can be analyzed individually or mixed in various combinations to create custom barcoded pools (BC Pools) that can be used in any experiment or assay (Fig. 1a, b).

Our own research is focused on breast cancer; hence, we applied the SunCatcher clonal barcoding method to several murine and human breast cancer cell lines. Breast cancer subtypes are defined based on hormone receptor expression and amplification of the Her2/neu oncogene and our parental cell lines were each originally derived from tumors of a distinct subtype. The cell lines we used include the McNeuA Her2-positive^41^, 4T1 triple-negative breast cancer (TNBC)^42^, and Met1 TNBC^43^ murine mammary carcinoma cell lines, as well as the human HMLER-HR TNBC cell line^44^. Only NBCs and BCs that were able to proliferate and form a colony were saved; not all single cells survived the subcloning process. We also discarded wells in which we observed either no cells, or more than one cell per well after the cell sorting/dilution plating step. Following the SunCatcher barcoding protocol, we typically achieved 30–40 different BCs per cancer cell line (Supplementary Table 1). For each cell line, we combined an equal number of each BC to generate breast cancer cell BC Pools (Supplementary Table 1).

Examining the clonal heterogeneity (i.e., BC composition) before, during, or after any in vitro or in vivo assay is achieved by first isolating genomic DNA and PCR-amplifying common barcode flanking regions. Several methods can then be used to deconvolute the barcode signals, including the previously described PRISM analysis method^29^, a specialized next-generation sequencing (NGS) assay, and/or a low-cost, rapid qPCR-based approach (Fig. 1b) that we developed. The deconvolution methods are described in detail below.

### Accurate qPCR-based barcode deconvolution method

**Fig. 2:**
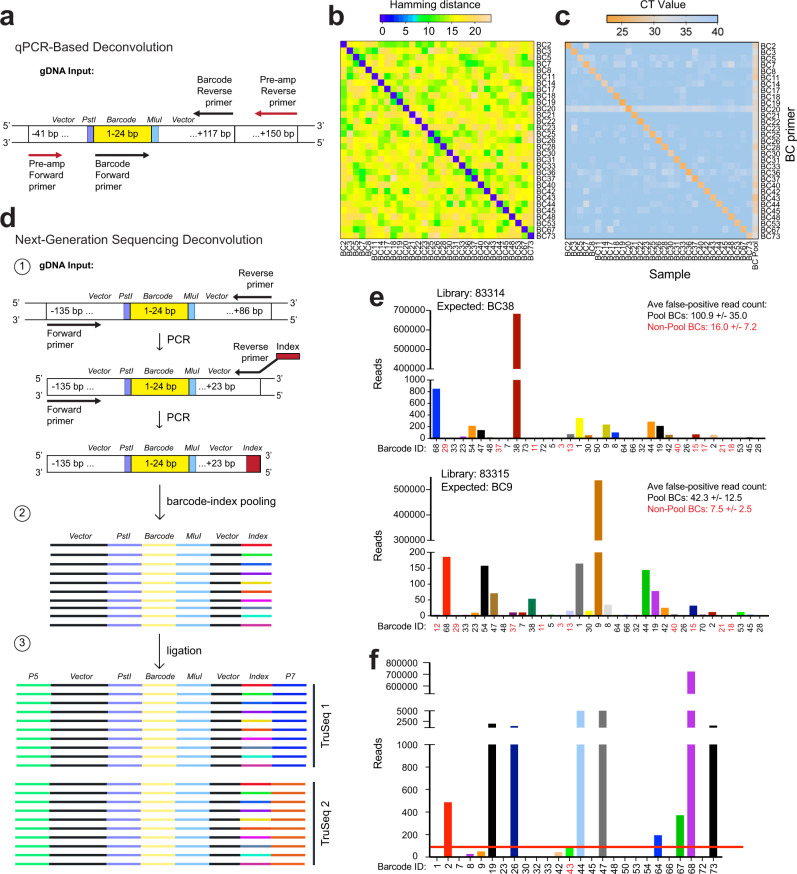
BC Detection by qPCR and next generation sequencing methods. **a** qPCR-based BC identification and quantification is achieved by subjecting sample gDNA to 2 rounds of PCR using pre-amplification primers (red, round 1) and BC-specific primers (black, round 2). **b** Heatmap showing hamming distance between all barcodes in the Met1 BC Pool. **c** Heatmap of qPCR cycle threshold (CT) values after testing each indicated barcode oligonucleotide primer against every Met1 BC population and the Met1-BC pool. **d** Multiplexing of gDNA samples for NGS is achieved in 3 steps. 1: Barcode regions in each gDNA sample are PCR-amplified using primers to universal barcode flanking sequences. To the resulting amplicons from each sample, one of 20 available unique 8-bp indexes is added downstream (3' end) of the barcode region via PCR. 2: Up to 20 different indexed amplicons (various colors) are pooled to generate a single barcode-index library. 3: Amplicons in each barcode-index library are ligated to Illumina adaptors. A universal P5 adaptor (green) is ligated upstream (5' end) and one of 24 available P7 adaptors (e.g., TruSeq1, blue and Truseq2, orange) is ligated downstream (3' end) of the barcode region. **e** Sequencing read counts from 2 HMLER-HR BC test samples. Each library corresponds to a single Illumina adaptor, and the expected barcode pair for each library is indicated. For each library, the average false-positive read count (±S.E.M.) per BC is shown for BCs in the HMLER-HR BC Pool (black) and for BCs not represented in the HMLER-HR BC Pool (red). All BCs that yielded a read count are represented. **f** Example of thresholding method for identifying BCs from experimental samples by NGS. Graph shows read counts for each barcode from an HMLER-HR BC Pool tumor. The only false positive read corresponds to BC43, which was used to set the false-positive threshold (red line). Barcodes with read counts below the threshold were discarded as sequencer noise. Source data are provided as a Source Data file.

We reasoned that qPCR would be a rapid, inexpensive method to identify and quantify barcodes from our BC Pools owing to the smaller library size of SunCatcher compared to traditional barcode libraries, which typically contain 10^4^–10^8^ barcodes^25,28,33,35^. We designed oligonucleotide primers to common barcode flanking sequences to enable a pre-amplification step (Fig. 2a; Supplementary Table 2). The pre-amplification step has several advantages. First, a separate PCR reaction is required to detect each barcode, so pre-amplification ensures sufficient genomic DNA (gDNA) input material, particularly from precious samples. Second, it adds a layer of specificity for BC detection above any potential background signal from the non-barcoded genome. Finally, it enriches all barcodes, including any very low abundance barcodes, thus enabling quantification of their contribution to any given sample.

We designed 24 bp oligonucleotides specific to each barcode sequence in order to detect each individual BC (Fig. 2a; Supplementary Table 2). To evaluate barcode similarity and the likelihood of barcode detection, we calculated the hamming distance between barcodes using the R library stringdist^45^ (https://github.com/petervangalen/BarcodeSimilarity). The minimum hamming distance between any two barcodes is 9 substitutions (Fig. 2b; Supplementary Fig. 1). Since our oligonucleotide primers are the reverse complement of the barcodes, the minimum hamming distance between any two primers is also 9 substitutions. In PCR analysis, this distance should prevent non-specific primer binding. In NGS analysis (described below), this distance is sufficient to prevent barcode collisions due to PCR or sequencing errors at one or a few positions.

To further ensure the specificity of our PCR detection method, we tested each individual barcode oligonucleotide primer against each individual BC population as well as the Met1 BC Pool. Every barcode was detected in the BC pool, while each oligonucleotide primer amplified only its specific barcode, as indicated by analyzing the C_t_ values for each reaction (Fig. 2c). Therefore, qPCR-based deconvolution offered a fast, inexpensive, and reliable method for accurately detecting individual barcodes from a polyclonal mixture of cells.

### Multiplex next-generation sequencing barcode deconvolution method

We sought to develop a next-generation sequencing (NGS)-based method of barcode detection in cases where multiplexing is desired. To do so, we first confirmed that BC sequences could be detected by Sanger sequencing using primers against the lentiviral barcode vector (Supplementary Fig. 2a). We validated that all 30 HMLER-HR BCs could be amplified and detected using Sanger sequencing (Supplementary Fig. 2b, c).

To maximize the number of samples per Illumina sequencer lane, we integrated multiple levels of multiplexing into our workflow. First, barcode regions in each gDNA sample are pre-amplified by PCR using primers to universal barcode flanking sequences (Fig. 2d, Supplementary Table 3). We introduce a second PCR step during which one of 20 unique indexes is added to the 3' end of the barcode amplicon (Fig. 2d, Supplementary Table 3), as previously described^46^. Up to 20 different indexed amplicons are then pooled in equal molar amounts to generate barcode-index libraries (Fig. 2d). Amplicons in each barcode-index library are then ligated to Illumina adaptors (Fig. 2d; Supplementary Table 4). Twenty-four uniquely indexed Illumina adaptors are commercially available, thereby allowing for the sequencing and deconvolution of up to 480 unique samples (20 barcode-index pairs x 24 indexed Illumina adaptors) in a single sequencing lane. Identification of the barcodes present within each sample thus requires deconvolution by first isolating all barcode reads associated with a single Illumina index and then isolating all barcode reads within a single Illumina index that are associated with the specific barcode-index associated with that sample.

We tested each indexed primer for its ability to successfully amplify the barcode region of gDNA extracted from HMLER-HR BCs, and then used Sanger sequencing to verify that the index had been incorporated. All 20 indexes could be added to the 3' end of barcode amplicons using PCR. We then tested whether our library preparation method could be used to accurately detect and deconvolute known barcode-index pairs that had been ligated into indexed Illumina adaptors. We used PCR to pair each of 17 indexes with two different barcode sequences to generate 17 barcode-index pools. We used input DNA from single BCs, so that a single known DNA barcode would be present in each sample. Additionally, we indexed DNA samples from the HMLER-HR BC Pool (which contains 30 DNA barcodes) with 3 separate indexes to examine a situation where a larger number of barcodes are associated with each index.

For Illumina library preparation, each of the 20 barcode-index pools was ligated to a uniquely indexed Illumina adaptor; the adaptors have identical P5 regions, and their P7 regions each contain a unique TruSeq Index (Fig. 2d). The 20 multiplexed samples were then mixed and run on a single lane of an Illumina MiSeq sequencer. To adjust for the low sequence diversity present in the sample, 40% PhiX was spiked into the sequencing reaction to increase the sample diversity^47^.

If ligation efficiency is low, one cannot use PCR to amplify the ligation products using primers against the Illumina P5/P7 adaptor sequences because every barcode in the pooled sample would get equally amplified. Therefore, as an alternative to ligation-based Illumina library preparation methods, we attached the Illumina adaptor regions to the amplicons being sequenced using a PCR-based library preparation method. To pursue this method, we designed a single forward PCR primer with complementarity to the barcode vector and 20 reverse primers that each contained a unique Illumina TruSeq index (Fig. 2d, Supplementary Table 4).

To evaluate whether this method enabled accurate identification of the expected barcode-Illumina index pairs, we generated a test library containing DNA samples from individual HMLER-HR BCs. The PCR reactions for each primer set were highly specific and efficient, generating a single, strong band of the expected length (Supplementary Fig. 3a). Analysis of the sequencing data revealed false barcode-Illumina index pairs for BCs that were in the HMLER-HR Pool as well as some BCs that were not in HMLER-HR pool in all samples (Fig. 2e, Supplementary Fig. 3b), indicating sequencer error^48^. Across all samples, the average false-positive read count per barcode sequence was 115.6 ± 45.0 for Pool BCs and 21.7 ± 8.8 for non-Pool BCs (Fig. 2e, Supplementary Fig. 3b). Importantly, the expected barcode-Illumina index pairs generated signal that was ~233–2,500-fold higher than the highest false positive signal, allowing for simple thresholding of true signal from background sequencer noise (Fig. 2e, Supplementary Fig. 3b).

We also developed a thresholding method for distinguishing true low-read count signal from false-positive signal for samples in which the expected BC identities are not known. In this case, we used HMLER-HR BC tumors. The HMLER-HR BC Pool contains 30 barcodes (Supplementary Table 1), which we designated, “expected barcodes”; we then selected 17 barcode sequences that are not represented in the HMLER-HR BC Pool as “unexpected” sequences. Thus, when analyzing the NGS data, we searched for the 30 expected BCs and the 17 unexpected sequences. When we sequenced the barcode amplicons prepared from gDNA extracted from an HMLER-HR BC Pool tumor, we detected only 1 unexpected sequence (BC43), which was used to set the false-positive threshold for that sample (Fig. 2f). For that tumor, 3 expected barcodes were below the threshold and thus discarded (Fig. 2f). Therefore, for each tumor sample analyzed by NGS, we set the highest unexpected barcode read count as the false-positive threshold; the “expected” BCs with read counts above that threshold are considered true signal, while those with read counts below the threshold are discarded. Hence, using these NGS approaches, BC sequences can be accurately identified and deconvoluted from tumor tissues in vivo.

### Tumorigenic properties are maintained during SunCatcher barcoding

**Fig. 3:**
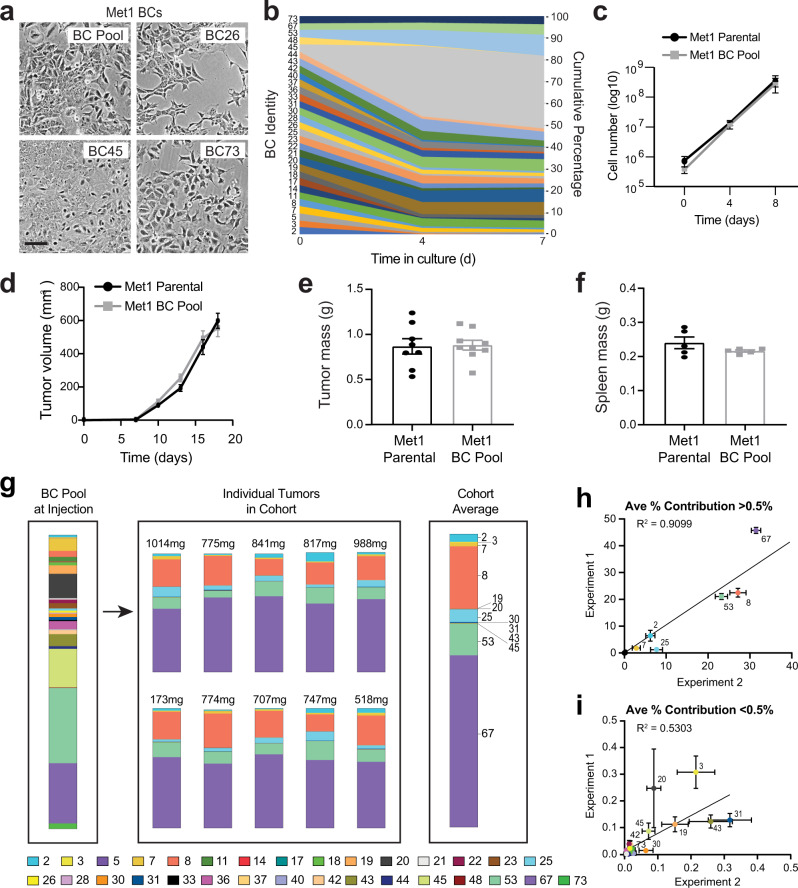
SunCatcher approach maintains tumorigenic properties and enables analysis of heterogeneity. **a** Phase contrast images of indicated Met1 BCs; scale bar = 100 μm. Images are representative of 2 independent observations. **b** Sand plot showing clonal composition (cumulative percentage) of Met1 BC Pool over 7 days (d) (2 passages) in vitro. **c** Growth of Met1 Parental (black; *Y* = 0.3360x + 0.1699) and Met1 BC Pool (gray; *Y* = 0.3562x + 0.4121) over 8 days in culture; *n* = 4 replicates per group; data are presented as mean values ± SD. **d** Growth of tumors from Met1 parental cells (*n* = 8 tumors) and Met1 BC Pool cells (*n* = 9 tumors) in FVB mice; *n* = 5 mice per cohort, data are presented as mean values ± SEM. **e**, **f** Mass (grams) of tumors (**e**) and spleens (**f**) from mice in experiment represented in (**d**); data are presented as mean values ± SEM. **g** Quantitative PCR assessment of barcode composition in the Met1 BC Pool at time of injection, in each of ten tumors (*n* = 5 mice) after 18 days, and average composition of all tumors in the cohort. Bars show indicated barcodes as a percent of total barcode signal (100%) within each sample; tumor mass is indicated above each bar. Key shows color code for each BC. **h**, **i** Comparison of the average representation of each BC in tumors for each of two independent experiments (*n* = 8 tumors for experiment 1; *n* = 6 tumors for experiment 2). BCs that constituted >0.5% of total BC signal (**h**) and <0.5% (**i**). Correlation coeff**i**cients (R^2^, simple linear regression) are shown; data are presented as mean values ± SEM. Source data are provided as a Source Data file.

It was important for us to understand whether the SunCatcher barcoding approach altered the complexity and important tumorigenic properties of the original parental cancer cell lines. To evaluate population complexity, we monitored cell morphology during the subcloning process. The BCs from each cell line exhibit a spectrum of cell morphologies, ranging from uniformly epithelial (e.g., Met1 BC45; Fig. 3a) to uniformly mesenchymal (e.g., Met1 BC26; Fig. 3a) phenotypes. Some BCs have mixed morphologies (e.g., Met 1 BC73 has both cobblestone epithelial and spindle-like mesenchymal phenotypes; Fig. 3a). We also confirmed that the BC Pools, like their respective parental lines, are morphologically diverse (Fig. 3a, Supplementary Fig. 4a).

We next evaluated BC Pool composition and growth over a period of 7 days (1 passage) in culture using our qPCR-based deconvolution method. The Met1 BC Pool composition shifted during the time course; while some clones expanded (e.g., BC45), no clones dropped below the limit of detection (Fig. 3b). Importantly, the proliferation rate of the Met1 BC Pool was nearly identical to that of the parental Met1 cell line (Fig. 3c), as was the HMLER-HR BC Pool relative to the HMLER-HR parental line (Supplementary Fig. 4b).

Results from similar analysis of the 4T1 BC Pool revealed the BCs that expanded and contracted during time in culture (Supplementary Fig. 4c), which were different from those identified in the Met1 BC Pool. Since the Met1 BC Pool and the 4T1 BC Pool share common barcode sequences, those results indicated that the BCs we identified as dominant in the BC Pools were unlikely the result of PCR primer/amplification bias.

It was most important for us to learn whether the SunCatcher “deconstruction-reconstruction” approach affected in vivo tumor biology. To do so, we orthotopically injected one cohort of FVB mice with 2.5 × 10^5^ Met1 Parental cells and another cohort with 2.5 × 10^5^ Met1 BC Pool cells (*n* = 5 mice per cohort) prepared after 7 days (1 passage) in culture. There were no significant differences between cohorts in tumor growth kinetics or in the final mass of tumors or spleens at the experimental end point (Fig. 3d–f). Likewise, we observed no significant difference in tumor growth or final tumor mass between the parental 4T1 cells and the 4T1 BC Pool (Supplementary Fig. 4d, e).

Collectively, these data revealed that the SunCatcher clonal barcoding approach preserved the heterogeneity as well as in vitro and in vivo growth kinetics of the original tumor cell lines from which they were derived. Moreover, subpopulations of just ~30 clones were sufficient to recapitulate phenotypic and functional properties of their respective parental populations.

### Clonal composition of Met1 TNBC tumors is reproducibly consistent

Having confirmed that the SunCatcher clonal barcoding approach retained the tumor growth kinetics of the parental cell lines, we asked how barcode composition compared across individual tumors of a given cohort and whether clonal composition is stochastic or consistent. To do so, we orthotopically injected 2.5 × 10^5^ Met1 BC Pool cells, prepared after 7 days (1 passage) in culture, into contralateral mammary fat pads of FVB mice (*n* = 10 tumors; *n* = 5 mice). As a control, we analyzed a sample from the injected cells that was collected when the cells were prepared for injection (“pre-injection” sample) and a sample taken after the last mouse was injected (“post-injection” sample) to determine if the BC composition shifted during the time (~2 h) that it took to perform all injections. The composition of the pre- and post-injection samples were nearly identical (Supplementary Table 5); hence, all mice in the cohort received the same input (Fig. 3g, Table 1).

**Table 1:** Met1 BC composition and clonal dynamics in vitro and in vivo.

	BC pool clonal dynamics in vitro			BC pool clonal dynamics in vivo			
BC	Pool admix (%)	Day 7 culture (%)	Fold Change	Injection (%)	Tumor Ave (%)	ST DEV	Fold change
2	3.2300	0.7459	−4.3302	0.7459	2.6848	2.0588	3.5993
3	3.2300	0.2218	−14.5635	0.2218	0.2247	0.1327	1.0133
5	3.2300	0.1089	−29.6563	0.1089	0.0058	0.0034	−18.9076
7	3.2300	4.3510	1.3471	4.3510	1.3084	0.6790	−3.3254
8	3.2300	2.0571	−1.5702	2.0571	21.2297	4.8215	10.3204
11	3.2300	1.5696	−2.0579	1.5696	0.0050	0.0057	−311.1084
14	3.2300	0.2405	−13.4321	0.2405	0.0002	0.0002	−1250.5905
17	3.2300	0.0207	−155.6942	0.0207	0.0003	0.0007	−65.5310
18	3.2300	1.0353	−3.1198	1.0353	0.0018	0.0035	−574.2797
19	3.2300	2.8757	−1.1232	2.8757	0.1045	0.0507	−27.5278
20	3.2300	8.3150	2.5743	8.3150	0.0693	0.0357	−120.0123
21	3.2300	0.4561	−7.0825	0.4561	0.0003	0.0005	−1820.1707
22	3.2300	1.2243	−2.6383	1.2243	0.0108	0.0111	−112.9192
23	3.2300	1.7757	−1.8190	1.7757	0.0417	0.0353	−42.5619
25	3.2300	0.7883	−4.0976	0.7883	4.3674	2.2510	5.5405
26	3.2300	0.6720	−4.8067	0.6720	0.0011	0.0020	−591.8461
28	3.2300	0.3914	−8.2532	0.3914	0.0010	0.0024	−391.3256
30	3.2300	1.0937	−2.9534	1.0937	0.0070	0.0083	−156.1097
31	3.2300	1.0464	−3.0867	1.0464	0.2874	0.1328	−3.6404
33	3.2300	0.3400	−9.5010	0.3400	0.0154	0.0187	−22.1346
36	3.2300	2.7291	−1.1836	2.7291	0.0404	0.0204	−67.5049
37	3.2300	0.0587	−55.0440	0.0587	0.0019	0.0043	−30.8490
40	3.2300	0.2769	−11.6657	0.2769	0.0198	0.0075	−13.9651
42	3.2300	1.3537	−2.3860	1.3537	0.0130	0.0149	−103.7583
43	3.2300	4.1184	1.2750	4.1184	0.1688	0.0679	−24.3918
44	3.2300	0.8892	−3.6323	0.8892	0.0060	0.0073	−147.9720
45	3.2300	13.1404	4.0682	13.1404	0.0603	0.0349	−217.8255
48	3.2300	0.1662	−19.4361	0.1662	0.0067	0.0069	−24.6266
53	3.2300	25.6185	7.9314	25.6185	10.7190	2.8135	−2.3900
67	3.2300	20.5118	6.3504	20.5118	58.5972	3.7022	2.8567
73	3.2300	1.8079	−1.7866	1.8079	0.0001	0.0003	−20788.2653

(Associated with data shown in Figs. 3 and 4). Clonal dynamics in vitro are represented as the fold change in BC composition of the Met1 BC Pool after 7 days in culture relative to initial admixture. Clonal dynamics in vivo are represented as the average fold change in BC composition of tumors at experimental end point relative to the Met1 BC Pool composition at the time of injection. The average contribution of each BC as a percent of total BCs in each resulting tumor (*n* = 10) and S.D. are indicated. Source data for average tumor composition are provided as a Source Data file.

After a growth period of 18 days, we harvested the tumors, isolated genomic DNA, and used our qPCR-based method to quantify the contribution of each barcode to each tumor. We observed similarity in BC composition across all 10 tumors in the cohort despite differences in tumor mass (Fig. 3g), suggesting that the tumor-forming capacity of this pre-clinical TNBC model is not stochastic. Given the consistency between tumors, we were able to represent the average BC composition for the entire cohort by calculating the average contribution of all detected BCs (Fig. 3g, Table 1).

We repeated the orthotopic tumor growth experiment to examine the reproducibility of the results between experiments. For BCs that constituted >0.5% of the total tumor composition, we observed concordance between results from the two different experiments (*R*^2^ = 0.9099; Fig. 3h). There was more variability within and between experiments for BCs that represented <0.5% of the total tumor BC composition (*R*^2^ = 0.5303; Fig. 3i). The reproducibility of our results suggested that there was phenotypic stability among the clones in vivo.

We also directly compared results from NGS deconvolution to those from qPCR deconvolution on the same tumors to determine whether there would be differences in sensitivity and/or accuracy between the two methods. For this comparison, we analyzed 6 tumors derived from the McNeu BC Pool (Ave tumor mass 280.3 mg ± 112.6 mg). We found significant concordance and correlation in barcode composition between methods (*R*^2^ = 0.975; *p* < 0.0001; Supplementary Fig. 4f). The qPCR-based detection method was more sensitive than NGS for low-abundance barcodes because NGS requires thresholding to distinguish between low-level false positive signals and signal generated by true, low-frequency barcode variants (Supplementary Fig. 4f).

These data indicated that intra- and inter-individual primary tumor formation and heterogeneity are notably consistent in the Met1 pre-clinical TNBC model. Such consistency enables the study of both the fittest and negatively selected clones to elucidate not only the properties that enable their formation, but also the vulnerabilities of those clones that were negatively selected.

### SunCatcher enables analysis of clonal dynamics in vitro and in vivo

It has been hypothesized that proliferation rates dictate clonal dynamics and that the most proliferative clones will dominate a tumor^4,49^. However, what provides the greatest selective pressure during disease progression has not been well elucidated. For example, it is not clear whether the most dominant clones in a tumor are inherently more proliferative or whether their emergence occurs via a selective process. As a first step toward answering that question, we analyzed the proliferation rate of each Met1 BC monoclonal population in vitro. Cells were counted every 72 h over a 9-day time course to calculate a population doubling value per day and BCs were ranked from highest to lowest doubling rate (Fig. 4a). The most rapidly proliferating clone, BC25, doubled at a rate ~2.3-fold that of the slowest proliferating clone, BC20 (Fig. 4a, Supplementary Table 6).

**Fig. 4:**
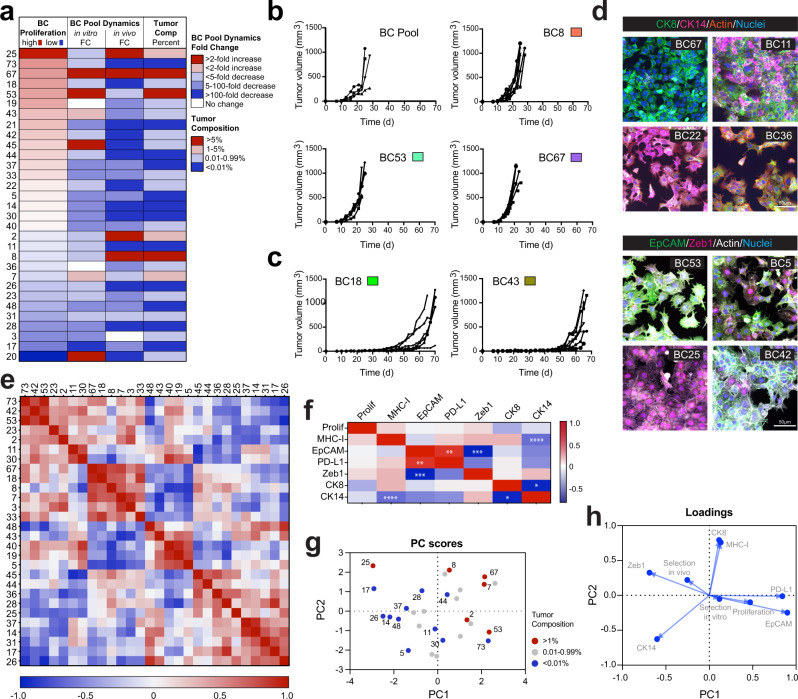
SunCatcher enables functional and phenotypic analysis of individual clones and clonal dynamics. **a** Functional analysis of Met1 BCs in monoculture and within the BC Pool. Column 1: proliferation rate (doubling time) of each BC over 9 days in culture, ranked from fastest to slowest. Each row represents data for the BC indicated in column 1. Column 2: Met1 BC Pool clonal dynamics in vitro, represented as fold-change (FC) in BC composition after 7 days (d) relative to initial admixture (data values shown in Table 1). Column 3: Met1 tumor clonal dynamics in vivo, represented as fold-change in BC composition at experimental endpoint relative to composition at time of injection (data values shown in Table 1). Column 4: average contribution of each BC as a percent of total BCs in each resulting tumor (*n* = 10). **b** Growth of Met1 BC Pool tumors (*n* = 4) and indicated individual BCs tumors (*n* = 6 per cohort). **c** Growth of indicated BC tumors (*n* = 6 per cohort). **d** Immunofluorescence images of indicated BCs stained for cytokeratin 8 (CK8, green), cytokeratin 14 (CK14, magenta), f-actin (red) (top), and epithelial cell adhesion molecule (EpCAM, green), zinc finger E-box-binding homeobox 1 (Zeb1) transcription factor (magenta), f-actin (white) (bottom). Nuclei were counterstained with DAPI (blue); Scale bars = 50 μm. Images represent 1 of 4 images per BC from 2 independent observations. **e** Pearson correlation matrix for individual Met1 BCs analyzed for: proliferation rate, CK8, CK14, Zeb1, EpCAM, programmed death ligand 1 (PD-L1), major histocompatibility complex type I (MHC-I) (associated with Supplementary Fig. 4 and Supplementary Tables 6–8). **f** Pearson correlation matrix for the 7 phenotypes assessed in vitro: proliferation rate, CK8, CK14, Zeb1, EpCAM, PD-L1, MHC-I (associated with Supplementary Tables 10, 11). Pearson correlation coefficients: CK8 v CK14 *p < 0.0215, 1-tailed, 90% CI; PD-L1 v EpCAM **p = 0.0096, 2-tailed, 90% CI; Zeb1 v EpCAM ***p = 0.0009, 1-tailed, 90% CI; MHC-I v CK14 ****p < 0.0001, 2-tailed, 90% CI. **g** Principal component (PC) analysis of BCs based on 9 parameters (proliferation, in vitro dynamics, in vivo dynamics, CK8, CK14, Zeb1, EpCAM, PD-L1, MHC-I) stratified by indicated percent composition of Met1 BC Pool tumors at experimental endpoint (associated with Supplementary Tables 9, 10). **h** Loadings plot from PCA shown in **g**. Source data are provided as a Source Data file.

We then examined how BC proliferation rates related to their contribution to the BC Pool in vitro. To do so, we calculated the fold-change in contribution of each BC to the BC Pool after 7 days (1 passage) in culture relative to the composition at the time the BC Pool was created (i.e., admixture of equal numbers of each BC) (Table 1). Four clones (BC20, BC45, BC53, and BC67) each underwent >2-fold expansion within the BC Pool in vitro*;* four clones (BC7, BC19, BC36, and BC43) exhibited modest increases or no change in their contribution over the 7-day period; the contribution of the remaining 23 clones decreased to varying degrees (Fig. 4a, Table 1). These analyses revealed that the relative proliferation rates of individual BCs did not predict their behavior within the BC Pool in culture. For example, the most proliferative clone, BC25, underwent a 4-fold decrease in contribution, while the least proliferative clone, BC20, underwent a 2.6-fold expansion within the BC Pool in culture (Fig. 4a, Table 1).

We assessed clonal dynamics in vivo by calculating the composition of BC Pool tumors at the 18-day end point and relating those values to the BC Pool composition at the time of injection (Table 1). Those analyses revealed four BCs that underwent expansion in vivo (BC2, BC8, BC25, and BC67), one BC that maintained its relative contribution (BC3), and 26 BCs that underwent negative selection to varying degrees (Fig. 4a, Table 1). Unlike the in vitro clonal dynamics in which BC25 underwent a 4.1-fold reduction, BC25 expanded 5.5-fold within the tumors (Fig. 4a, Table 1). In contrast, BC20, which had expanded 2.6-fold in culture, experienced a 120-fold decrease in contribution to the tumors (Fig. 4a, Table 1). While the in vivo behavior of these two clones (BC20 and BC25) reflected their relative in vitro monoclonal proliferation rates, those same trends were not observed in all clones. For example, BC73, the second-most proliferative clone in vitro, underwent negative selection within the tumors, with a ~20,000-fold reduction in contribution (Fig. 4a, Table 1).

Three clones together consistently comprised ~90% of total BC signal detected in the tumors at the experimental end point: BC8 (21.2 ± 4.8%), BC53 (10.7 ± 2.8%), and BC67 (58.6 ± 3.7%) (Fig. 4a; Table 1). BC53 and BC67 were among the most dominant clones in the BC Pool at the time of injection (25.6% and 20.5%, respectively) and had similar growth rates as monoclonal cultures and within the BC Pool in culture (7.9- and 6.4-fold increases, respectively) (Fig. 4a, Table 1). Hence, despite their similar behavior in culture, the contribution of BC53 contracted ~58% while BC67 expanded ~185% in vivo (Fig. 4a, Table 1). Therefore, to determine whether the outcomes of BC53 and BC67 in the resulting tumors were the consequence of clonal dynamics or their own inherent tumor growth capacity, we orthotopically injected BC53 and BC67 into mice as monoclonal populations. Each of these two clones displayed ~10-day latency and rapid growth kinetics, which were similar to each other and that of the BC Pool (Fig. 4b). Those results suggested that BC53 was particularly susceptible to clonal dynamics in vivo and BC67 appeared to be unchanged by in vivo selection pressures.

In contrast, BC8 comprised only ~2% of the BC Pool at the time of injection (Table 1) and had a relatively low proliferation rate in vitro (Fig. 4a, Supplementary Fig. 5a). Nevertheless, the relative contribution of BC8 expanded ~10-fold in vivo (Fig. 4a, Table 1). When injected as a monoclonal population, BC8 displayed relatively short latency (~10–12 days) and rapid growth kinetics, reflective of the BC Pool tumors (Fig. 4b). Those results suggested that BC8 experienced positive selective pressure in vivo, causing it to become one of the most rapidly proliferating clones.

Some clones appeared to undergo negative selection in the polyclonal setting, either in vitro or in vivo. For example, BC45, which had a relatively high proliferation rate and represented a dominant clone within the BC Pool at time of injection, represented only <0.1% of the resulting tumors (Fig. 4a; Table 1). Likewise, other clones, such as BC14, BC17, BC21, and BC73, diminished to only <0.0004% of the tumors (Table 1).

One of the most proliferative clones in vitro, BC18, underwent a 3-fold reduction in relative contribution to the BC Pool in culture and a 574-fold reduction in vivo (Fig. 4a; Table 1). Another clone with a high proliferation rate in vitro, BC43, comprised ~4% of the BC Pool at time of injection yet was diminished to <0.2% of the resulting tumors (Fig. 4a, Table 1). Those 2 clones, BC18 and BC43, were each capable of forming monoclonal tumors after a latency period of ~40–50 days, and with variable incidence (Fig. 4c). Importantly, those results suggested that the contribution to disease progression of clones with inherent tumorigenic potential after a long latency period (e.g., BC18 and BC43) might have been overlooked because ethical size limitations of the BC Pool tumors dictated end points prior to emergence of those clones from latency.

Collectively, these results suggested that clonal dynamics and/or environmentally driven selection pressures ultimately influence the fate of certain BCs, while the inherent proliferation of other BCs is not impacted in the polyclonal setting. Although these concepts have been previously established^17,19,50^, SunCatcher enabled us to identify and analyze the specific clones within heterogeneous tumors that are influenced by such dynamic processes in vitro and in vivo.

### SunCatcher enables identification of phenotypes associated with outcomes

One of the advantages of the SunCatcher clonal barcoding approach is the ability to obtain useful information from analyses of live cells of interest, regardless of their outcome in any given experiment. To test that concept, we designed experiments to determine whether individual BC phenotypes in vitro correlate with their fate in vivo, namely, their contribution to the resulting Met1 BC Pool tumors. We therefore performed single-cell phenotyping of each Met1 BC for factors known to impact tumorigenesis and disease progression^51^. We focused on markers of stratified epithelium (cytokeratin 8 and 14), epithelial-mesenchymal transition (zinc finger E-box-binding homeobox 1 (Zeb1) and epithelial cellular adhesion molecule (EpCAM)), and immune regulation (major histocompatibility complex (MHC-I) and programmed death-ligand 1 (PD-L1)).

Immunocytochemical analysis of cytokeratin 8 (CK8, “luminal”), cytokeratin 14 (CK14, “basal”), and nuclear Zeb1 staining revealed varying levels of expression among the clones for each factor (Supplementary Fig. 5a, Supplementary Table 6). Some BCs expressed a single cytokeratin (e.g., BC67 expressed CK8; BC22 expressed CK14) (Fig. 4d). Other BCs contained cells that stained for either CK8 or CK14 to varying extents (e.g., BC11), and some BCs (e.g., BC36) were comprised of individual cells that expressed both CK 8 and CK14 (Fig. 4d). Similarly, co-staining for EpCAM and Zeb1 revealed BCs that almost exclusively expressed either nuclear Zeb1 (e.g., BC25) or EpCAM (e.g., BC53) (Fig. 4d). Most BCs (e.g., BC5 and BC42) expressed both factors to varying extents (Fig. 4d). Hence, despite their clonal origin, some BCs gave rise to phenotypically diverse populations.

We also performed single-cell phenotyping by evaluating cell-surface expression of EpCAM, PD-L1, and MHC-I for each BC by flow cytometry. In this case, we quantified baseline levels of PD-L1 and MHC-I in the absence of IFNγ stimulation. BCs were then ranked according to their mean fluorescence intensity for each factor and again, we observed varying levels of expression among the BCs (Supplementary Fig. 5b, Supplementary Table 6).

We next asked how the BCs relate to one another with respect to the seven in vitro hallmark phenotypes that we analyzed (CK8, CK14, Zeb1, EpCAM, MHC-I, PD-L1, and relative proliferation rate; Supplementary Fig. 5a, b). To do so, Z-scores were generated for each biological parameter for each BC (Supplementary Table 7). We then generated a Pearson correlation matrix to visualize the relationships between clones (Fig. 4e, Supplementary Table 8). The matrix revealed significant heterogeneity among all the BCs as well as clusters of BCs that are significantly correlated with one another with respect to the 7 biological features (Fig. 4e, Supplementary Table 8). We discovered cases in which phenotypically dissimilar clones shared common fates in vivo. For example, BC25 and BC53 are significantly anti-correlated (*r* = −0.64; Fig. 4e, Supplementary Table 8) yet both were dominant clones in the tumors (Fig. 4a). Additionally, we observed that some clones were highly similar phenotypically, yet their fate diverged in vivo. For example, the most highly correlated clones, BC53 and BC73 (*r* = 0.95; Supplementary Table 8), had different outcomes in vivo; BC53 comprised 10.71% of the resulting tumors compared to BC73, which comprised <0.01% of the tumors (Table 1).

We next determined how closely correlated CK8, CK14, Zeb1, EpCAM, MHC-I, PD-L1, and proliferation rate were across all Met1 BCs. We confirmed the expected significant anti-correlations between CK8 and CK14^52^ (*R*^2^ = 0.5923, *p* = 0.0215). Nuclear Zeb1 and cell-surface EpCAM expression were significantly anti-correlated (*R*^2^ = 0.98782, *p* = 0.0009) (Fig. 4f, Supplementary Table 11). Moreover, PD-L1 and EpCAM expression were significantly correlated (*R*^2^ = 0.2311, *p* = 0.0096) (Fig. 4f, Supplementary Table 11), and cell-surface expression of MHC-I was significantly anti-correlated with CK14 expression (R^2^ = 0.7534, *p* < 0.0001) (Fig. 4f, Supplementary Table 11). None of the phenotypic parameters was significantly correlated with in vitro proliferation (Fig. 4f).

To reduce dimensionality and visualize trends in the data, we stratified tumor composition into 3 cohorts (>1%, 0.01–0.99%, <0.01%) and performed principal component analysis (PCA) on the 9 variables that we had measured (CK8, CK14, Zeb1, EpCAM, MHC-I, PD-L1, proliferation rate, in vitro fold change in contribution to the BC Pool, and in vivo fold change in contribution during tumor progression) (Supplementary Table 9). PCA revealed separation between those BCs that comprised the highest (>1%) and lowest (<0.01%) proportion of the tumors (Fig. 4g), with two notable outliers: BC25 and BC73 (Fig. 4g). To understand which variables were most influential in the PCA patterns, we visualized the PC loadings (Fig. 4h). That analysis revealed that PC1 is most positively influenced by EpCAM (*r* = 0.90) and PD-L1 (*r* = 0.84) expression and negatively impacted by Zeb1 (*r* = −0.69) and CK14 (*r* = −0.60) expression (Fig. 4h, Supplementary Table 10). PC2 is most associated with CK8 (*r* = 0.79) and MHC-I (*r* = 0.76) and is negatively associated with CK14 (*r* = −0.63) (Fig. 4g, Supplementary Table 10).

Collectively, these results revealed that the ability to analyze individual clones in various contexts and at different time points, yields important information about biological properties that associate with outcomes. Moreover, these findings provided proof-of-concept that the ability to study clones that were eliminated or otherwise not selected during our experimentation yielded important information.

### Identification and quantification of early spontaneous metastasis

Several studies, including those of breast cancer patient xenografts, provide evidence that metastases are derived from subclones present at low frequency in the primary tumor^39,53–56^. However, early spontaneous metastases from orthotopic sites are often difficult to detect using conventional experimental detection methods, such as bioluminescence of luciferase-labeled tumor cells, for a variety of reasons^57^. We therefore tested the sensitivity of SunCatcher to detect and identify clones that comprise spontaneous metastases that could then inform future functional analyses of those metastatic clones.

We injected GFP-Luciferase-labeled Met1 parental cells^17^ either orthotopically or intravenously into cohorts of mice (*n* = 9) and analyzed the lungs ex vivo 21 days later. We did not detect lung metastasis by IVIS imaging or by visual observation in the mice that had been orthotopically injected (Supplementary Fig. 6a, b). Lungs of mice that had been injected intravenously only had detectable signals in 2/9 mice, although visual observation showed overt metastatic nodules in the majority of the lungs (Supplementary Fig. 6a, b). Those findings were in line with a recent report demonstrating that immunogenicity of fluorescent proteins used to tag cells often hinders pre-clinical metastasis research in immune competent models^57^.

**Fig. 5:**
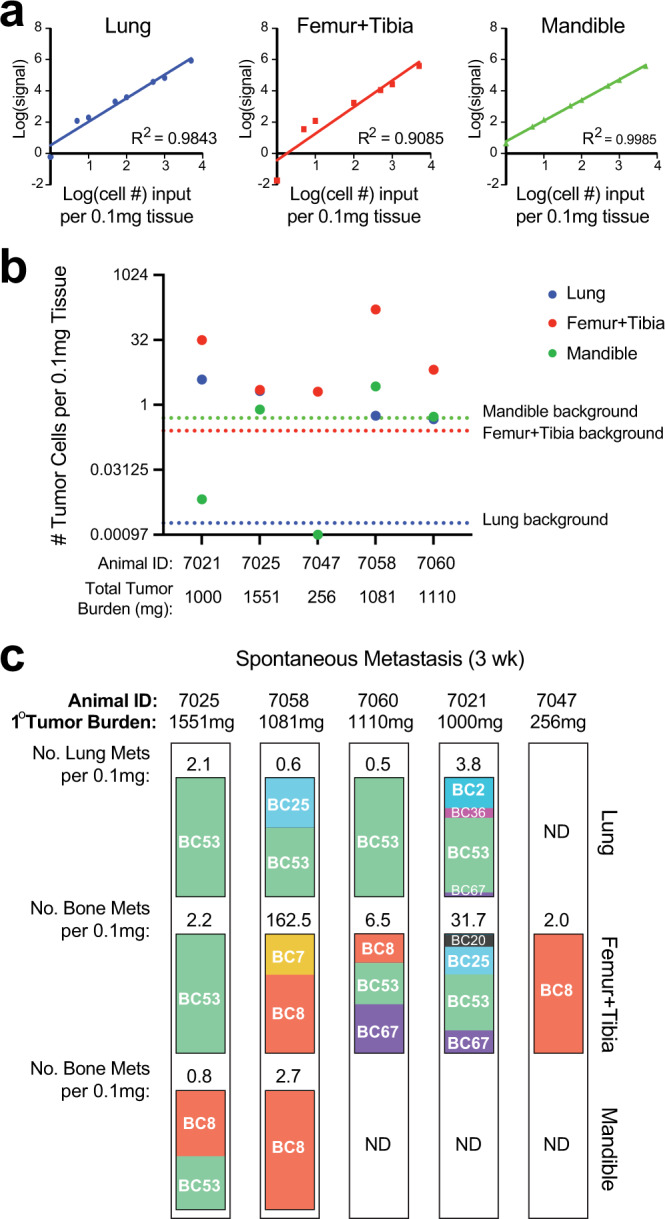
SunCatcher enables identification and quantification of early spontaneous metastasis. **a** Calibration curves were generated for indicated tissues by serially diluting known amounts of barcoded tumor cell gDNA into a fixed amount of normal tissue gDNA. From left to right: lung, long bones (from femur and tibia), mandible. **b** 2.5 × 10^5^ barcoded Met1 tumor cells were injected bilaterally into the mammary fat pads (*n* = 5 animals) and tumors were allowed to grow for 21 days, at which point tissues were harvested and metastasis burden was calculated. Dashed lines indicate the background signals from each indicated tissue type. Tissues with signal above the background were considered positive for metastasis and estimated tumor cell number per 0.1 mg tissue was calculated based on the calibration curve for that specific tissue. **c** Barcode composition analysis on tissues with positive metastasis signal. Bars represent percent of total barcode signal (100%) within each sample. Also shown are mouse identities, total primary tumor burden for each animal, and estimated numbers of metastases per tissue; N.D., not detected. Source data are provided as a Source Data file.

Given the sensitivity of SunCatcher barcode detection and reproducibility of results, we asked whether we might detect early Met1 BC Pool metastases from orthotopic primary tumor sites using our qPCR-based detection method. We first devised a method that would enable us to extrapolate numbers of metastatic cells based on the total barcode qPCR signal obtained from a fixed mass of tissue (see Methods). Our method also enabled thresholding to distinguish real signal from background signal for each tissue; consequently, we were able to reproducibly detect 1 cell per 0.1 mg of tissue (Fig. 5a).

To evaluate spontaneous metastasis, we injected the Met1 BC Pool into contralateral mammary glands and examined visceral and skeletal tissues 21 days later. Total primary tumor burden was similar between 4/5 mice (mean 1185.5 mg; range 1000–1551 mg), and only one small tumor (256 mg) formed in one mouse (Fig. 5b). Mice were otherwise healthy, and we did not observe any overt metastases upon visual examination of tissues. However, barcode deconvolution by qPCR indicated that the lungs, long bones, and mandibles consistently contained barcode signal (Fig. 5b).

Among the tissues in which barcodes were detected, the leg bones (femur and tibia combined) had the greatest relative numbers of spontaneous metastases, regardless of the primary tumor burden, as 100% of the mice had a barcode signal above the threshold (Fig. 5b). Lung metastasis was detected in 4/5 mice, and metastasis to the mandible was detected in 2/5 mice (Fig. 5c).

BC53 was a dominant clone in 100% of mice that had lung metastasis and the only clone that was represented in more than 50% of lung metastasis (Fig. 5c). In the leg bones (femur and tibia), the inter-individual composition of BC metastases was slightly more variable and diverse (Fig. 5c). For example, unlike in the lung, BC53 was detected in 60% of the mice with metastases to the leg bones (Fig. 5c). The mice with the lowest tumor burden (mouse 7047; 256 mg) and highest tumor burden (mouse 7025; 1551 mg) each had clonal leg bone metastases (BC8 and BC53, respectively; Fig. 5c). BC8 and BC53 were the only barcodes detected in the mandibles, and in that tissue, BC8 was the dominant clone (Fig. 5c).

We were also able to assess intra-individual differences in composition across different metastatic sites. Mouse 7025 displayed relative consistency across all tissues, with BC53 clonal metastasis in the lung and leg bones, and BC8 appearing only in the mandible (Fig. 5c). Remarkably, mouse 7047 had the lowest primary tumor burden yet had BC8 clonal metastasis in the leg bones (Fig. 5c). Those results suggested that BC8 is particularly amenable to bone metastasis.

Collectively, these results indicated that SunCatcher offers a functional barcoding approach to study clonal dynamics during metastatic progression. Importantly, the qPCR-based detection method enabled us to identify and quantify metastases even at very early stages from orthotopic sites.

## Discussion

We developed the SunCatcher clonal barcoding method to enable functional analysis of the subclones that comprise complex, heterogeneous populations of cells. With SunCatcher, each clone is specific to a single barcode; there is no chance that a clone contains more than one unique barcode sequence. Therefore, a potential complication of those barcoding methods that rely on infecting a heterogenous population of cells with a large barcode library is avoided. While the generation of individual clones is initially labor-intensive, once the NBC and BC stocks are established, they can be resourced indefinitely. A significant benefit of the qPCR-based BC detection method is that it is rapid, sensitive, reliable, and inexpensive. The advantage of the NGS-based deconvolution method is that it allows for multiplexing if larger barcode libraries are desired.

We found that pools of just ~30 heterogeneous clones were sufficient to recapitulate tumor growth kinetics of the respective parental population. Additionally, a limited analysis revealed some of the phenotypes that associated with BC representation-both positive and negative selection-in the resulting tumors. Nevertheless, whether we captured the entirety of the heterogeneity represented in the original parental population is not clear. Finding ways to estimate the number of clones required to represent the heterogeneity of a complex parental cell line could be an important consideration when applying SunCatcher to other studies. The Suncatcher system should easily enable unbiased transcriptional profiling or more specialized functional assays to answer those remaining questions. However, whether clonal phenotypes were altered by the subcloning process cannot be answered using the SunCatcher approach alone.

Our single-cell analyses of BC cell phenotypes exposed additional benefits of the SunCatcher system. First, our clonal approach enables one to trace clonally related cells, even as the inherent plasticity of those cells gives rise to phenotypic heterogeneity. For example, we found that some BCs were comprised of both CK8+ and CK14+ cells. A common experimental approach is to sort cells based on an expression marker of interest (e.g., CK8 and CK14) and then perform functional analyses on the sorted populations to identify differences in their behavior or responses to treatment. Without using molecularly tagged cells in such an experiment, the clonal relationship between some of the CK8+ and CK14+ cells would not be revealed. Second, because stocks of individual BCs are retained, SunCatcher uniquely enables one to not only identify but also isolate the common ancestor of cells that might otherwise appear to be unrelated by other analysis methods. Third, unlike SunCatcher, most traditional barcoding approaches, in which a heterogeneous cell population is infected with a barcode library, do not enable retrospective isolation and functional analysis of the clone of origin of cells with relevant phenotypes (e.g., tumor formation, metastatic progression, or drug responses). The SunCatcher approach would thus enable future identification of genetic and epigenetic mechanisms that drive functional differences between clones.

Using SunCatcher, we identified clones that constituted a very minor proportion of the BC Pool tumors at the experimental end point yet had the potential to form rapidly growing tumors with variable incidence after a long latency period (~30–40 days longer than the BC Pool) when injected as a pure clonal population (e.g., BC18 and BC43). Further work is required to understand whether the low abundance of those clones within the heterogeneous primary tumors is a result of their inherent latency, and/or whether they are inhibited by signals derived from other clones (or the microenvironment) in the pooled setting vivo. Pre-clinical end points are dictated by maximum humane tumor size (1 cm in our case) and so analysis of the BC Pool tumor composition could not extend beyond that end point. Our findings therefore raise questions about the fate of those minor clones if our experimental time points were extended or if those clones represented residual disease after surgical resection or drug treatment. In fact, our findings are in line with a report investigating melanoma stem cells, which demonstrated that all patient-derived tumor cells had tumor-initiating potential if given sufficient time (very protracted in some cases) following injection^58^. Additional reports, including our own, have indeed demonstrated variable engraftment and growth rates of single-cell-derived clones^17,39,56^.

In our experience, we were previously unable to detect early metastases in our pre-clinical models using conventional detection methods. Moreover, we had assumed that our Met1 cells were unable to spontaneously metastasize to distant organs before primary tumors reached ethical end point. Our detection of spontaneous metastases in lungs and skeletal tissues after only 3 weeks are in line with numerous studies that have detected circulating and disseminated tumor cells in the early stages of cancer progression in both preclinical models and in patients^59–62^. We envision that SunCatcher could provide valuable information about the properties of individual disseminated tumor cells that make them more or less threatening than their counterparts and aid in the identification of therapeutic targets that could prevent their progression.

A current limitation to SunCatcher is that we cannot directly isolate discreet live BC cells from the BC Pool tumors. The ability to do so could ostensibly reveal important information about BC gene expression and phenotypic plasticity during disease progression. This limitation may be overcome by incorporating BC identification into single cell analysis technologies. Nevertheless, we found that one of the more important benefits of SunCatcher is the ability to identify and functionally analyze clones that are negatively selected during experimentation. That ability could be especially important for evaluating drug responses and identifying critical cell vulnerabilities. The ability to study “super responders” could lead to development of therapeutic approaches that prevent disease progression.

## Methods

Mass General Brigham provided institutional approval of biosafety protocol 2016B000089 and provided ethical oversight.

### Cell lines

The Met1 TNBC cell line, which was originally generated from a spontaneously arising tumor in an FVB/N-Tg (MMTV-PyVmT) mouse^43^, was a gift from J. Joyce (University of Lausanne) with permission from A. Borowski (UC Davis School of Medicine) and maintained in DMEM containing 10% fetal bovine serum, 100 U/mL penicillin-streptomycin, and 4 mM glutamine, as previously described^17^. The 4T1 TNBC cell line, which was derived from a spontaneously arising tumor in a Balb/c mouse^42^, was provided by F. Miller (Wayne State University School of Medicine) and maintained in DMEM containing 10% fetal bovine serum, 1x MEM non-essential amino acids solution (Gibco), 100 U/mL penicillin-streptomycin, and 4 mM glutamine. The McNeuA HER2+ cell line, which was originally derived from a spontaneously arising breast tumor in an MMTV-neu transgenic mouse, was provided by Michael Campbell (University of California, San Francisco) and maintained in DMEM (Gibco) supplemented with 10% fetal bovine serum (FBS) and 100 U/mL penicillin-streptomycin (Gibco) at 37 °C with 5% CO_2_^41^. Human HMLER-hygro-H-*ras*V12 (HMLER-HR) cells^44,63^ were derived from human mammary epithelial cells (HMEC) originally obtained from ATCC. were cultured in advanced DMEM/F12 (Gibco) supplemented with 5% calf serum (CS, HyClone), 0.1% hydrocortisone (Sigma Aldrich), and 100 U/mL penicillin-streptomycin (Gibco) at 37 °C with 5% CO_2_. 293 T cells were cultured in DMEM (Gibco) supplemented with 10% FBS (Gibco) and 100 U/mL penicillin-streptomycin (Gibco) at 37 C with 5% CO_2_. All cell lines were tested and were negative for mycoplasma (Lonza) and short tandem repeat analysis verified the identity of mouse (Bioassay Methods Group, National Institute of Standards and Technology) and human (Promega GenePrint 10 System) cells.

### Lentiviral barcode vectors

Lentiviral vectors were a generous gift from Dr. Todd R. Golub. Barcode sequences, originally developed by Tm Bioscience (Toronto, Ontario, Canada, http://www.tmbioscience.com), are described in the Luminex FlexMAP Microspheres Product Information Sheet (http://www.luminexcorp.com) and provided as Lentiviral vectors^29,40^. 293 T cells were transfected with 2.5 μg lentiviral vector barcode DNA, 2.5 μg pCMV-dR8.2 dvpr (Addgene plasmid 8455), 1 μg pCMV-VSVG (Addgene plasmid 8454) in Opti-MEM Reduced Serum Medium (Gibco) with Fugene HD (Promega) or FuGENE6 (Roche Corporation). Viral supernatants were collected 48 and 72 h after transfection, filtered through a sterile 0.45-μm syringe filter (VWR), and stored at −80 °C.

### SunCatcher barcoding protocol

Single cells were isolated from cell lines by either FACS or plating 0.5 cells/well in 96-well plates. We verified that each well contained a single cell by phase microscopy under 40x magnification and discarded wells that contained more than one cell. Each single-cell clone was expanded, and these cells were designated non-barcoded clones (NBCs). Aliquots of 5 × 10^5^ cells of each NBC were prepared in appropriate culture medium with 10% DMSO and stored in cryovials in liquid nitrogen.

For barcoding, each NBC population was thawed into a 6-well plate and infected with a unique barcode-containing lentivirus supernatant in appropriate medium with Polybrene (Sigma Aldrich) at a concentration ranging from 1:200 to 1:50. After 24 h, virus was washed out and replaced with fresh medium with 10 μg/ml blasticidin (Sigma). After 3 days, cell numbers were counted in each condition and those with an infection rate of <10% (number of infected cells remaining in selection media / infected cells grown in regular growth media) were selected. Single cells were then isolated from the infected populations by plating 0.5 cells/well in 96-well plates and then expanded to generate multiple individual clonal populations with each barcode. A single clonal population was selected at random for each unique barcode, which was referred to as the barcoded clonal population (BC). Aliquots of 5 × 10^5^ cells of each BC were prepared in an appropriate culture medium with 10% DMSO and stored in cryovials in liquid nitrogen.

For each cell line, equal numbers of every BC were mixed to form a BC Pool. Aliquots of 5 × 10^5^ cells of each BC Pool were prepared in appropriate culture medium with 10% DMSO and stored in cryovials in liquid nitrogen.

### Genomic DNA preparation from cells and tissues

Cultured cells were detached using 0.25% trypsin, pelleted, and resuspended in Buffer AL (Qiagen). Genomic DNA (gDNA) was extracted using a QiaAmp DNA Mini Kit (Qiagen) according to manufacturer’s instructions. Tumors and other tissues were flash frozen in liquid nitrogen and pulverized using a mortar and pestle. 25 mg of tissue was used for DNA isolation according to the manufacturer’s protocol (Qiagen). Femurs and mandibles were immediately dissected and cleaned to remove muscle and connective tissue and were then flash frozen and pulverized (preparations included marrow). 25 mg of bone preparations were used for DNA isolation according to the manufacturer’s protocol (Qiagen). The DNA concentration was quantified using a NanoDrop 8000 Spectrophotometer (Thermo Fisher).

### qPCR-based barcode identification and analysis

To detect barcodes, gDNA was first pre-amplified by preparing 50 μl reactions containing 500 ng gDNA, OneTaq 1x MasterMix (New England Biolabs), and 0.4 μM of each F/R pre-amplification primer to common flanking sequences (Forward: 5'-CGATTAGTGAACGGATCTCG-3'; Reverse: 5'-CCGGTGGATGTGGAATGTG-3') (Supplementary Table 2). The following PCR program was used to amplify the template DNA: 94 °C for 30 s, followed by 40 cycles of 94 °C for 30 s, 52 °C for 30 s, and 68 °C for 30 s; a final extension was performed at 68 °C for 5 min. The PCR products were purified by a Monarch® PCR & DNA Cleanup Kit following the manufacturer’s instructions. Purified PCR products were eluted in 50 μl water, and the DNA concentration was determined using a NanoDrop 8000 Spectrophotometer (Thermo Fisher). To quantify the abundance of specific BCs in any given sample, the purified pre-amplification PCR products were analyzed by qPCR, for which each barcode sequence^40^ was used as the forward primer and 5'-CCACTTGTGTAGCGCCAAG-3' was used as the universal reverse primer (Supplementary Table 2). Each sample was tested for all barcodes by setting up an individual reaction for each barcode primer set for each sample. Each qPCR mixture contained 0.001 ng of purified PCR product, 1 μM of each F/R primer, and 1X iTaq™ Universal SYBR Green Supermix (Bio-Rad) in a 10-μL reaction volume. To ensure there is no cross-contamination of gDNA between samples, all sample handling is performed in a benchtop containment hood and control water samples are carried through the entire protocol. The following program was used: 50 °C for 2 min and 94 °C for 10 min, followed by 40 cycles of 94 °C for 30 s and 60 °C for 30 s. Dissociation curves were collected after qPCR, and quality control of qPCR signals was performed to ensure that there was a single peak, thus indicating that amplification of a single barcode occurred in each reaction. The Ct values acquired from the qPCR run were converted to arbitrary units (AU, (1): AU = 2,000,000,000*EXP (−0.69*Ct value)), and the values of the individual barcodes in every sample were summed. The percentage of each barcode present in the sample was calculated by dividing the individual barcode signal by the sum of all the barcode signals.

### Barcode identification and analysis by next-generation sequencing

From gDNA preparations, the barcode region of the lentiviral barcode vector was amplified using either primer set JO primer F/R (F: 5'-TGGAGCATGCGCTTTAGCAG; R: ATCGTTTCAGACCCACCTCC-3') or indexed primer sets (F: JH p05: 5'-ATC GTT TCA GAC CCA CCT CCC-3'; R: JH p11-14 and JH p31-46, Supplementary Table 3). PCR mixtures contained 250 ng DNA, OneTaq 2x MasterMix (New England Biolabs), and 10 μM each of F/R primers. The following PCR program was used to amplify the template DNA: 94 °C for 30 s, followed by 30 cycles of 94 °C for 20 s, 54 °C for 30 s, and 68 °C for 20 s; a final extension was performed at 68 °C for 5 min. PCR products were purified using Agencourt AMPure XP beads (Beckman Coulter) according to the manufacturer’s protocol, and purified DNA was eluted in 20–40 μl water.

Ligation-based Illumina library preparation was carried out by the Harvard Biopolymers Facility using an Integex Apollo 324 PrepX ILM kit using Kappa reagents according to manufacturers’ protocols. For PCR-based Illumina library preparation, samples that had been amplified using one of the barcode-indexed primer sets were subjected to a second PCR using an Illumina-indexed primer set with regions that were complementary to the barcode lentiviral vector regions (F: JO p50, R: JO p65-p88; Supplementary Table 4). PCR mixtures contained 100 ng DNA, OneTaq 2x MasterMix (New England Biolabs), and 10 μM each of F/R primers. The following PCR program was used to amplify the template DNA: 94 °C for 30 s, followed by 15 cycles of 94 °C for 20 s, 54 °C for 30 s, and 68 °C for 20 s; a final extension was performed at 68 °C for 5 min. PCR products were purified using Agencourt AMPure XP beads (Beckman Coulter) according to the manufacturer’s protocol, and purified DNA was eluted in 20–40 μl water.

### Sequencing data analysis

Barcode-specific PCR products with Illumina adaptors were mixed in equimolar ratios and sequenced on an Illumina MiSeq at a depth of 20–30 × 10^6^ reads. First, reads that did not contain the barcode adaptor sequence ((2): (8 base index)-(17 bases)-ACGCGT-(24 base barcode)-CTGCAG) were filtered out. Samples were grouped based on the 8-base index, and a read count table was generated based on all 24-bp barcode sequences present in each read. To identify barcodes, each barcode on the read-count table was compared to the 24-base barcode sequences in the barcode library. The script for identifying barcode sequences is available at https://github.com/petervangalen/BarcodeSimilarity/blob/master/McAllister_SunCatcher/match-barcode.py. A receiver operating characteristic (ROC) curve was generating by classifying the 24-bp barcodes as “True” (present in the barcode library) or “False” (absent from the library). Thresholding was performed based on the read count that maximized the area under the curve (AUC) from the ROC curve.

### Animal experiments

All animal studies were conducted in accordance with regulations of the Institutional Animal Care and Use Committee of the Brigham and Women’s Hospital (protocol no. 2017N000056). Mice were subject to 12-h day/night cycles and were housed at 21–23 °C with an ambient humidity of 40–60%. For all experiments, 6–8-week-old female FVB/NJ and BALB/cJ mice were purchased from Jackson Laboratory; mice were 8–9 weeks of age at the time of injection. For orthotopic injections, 2.5 × 10^5^ Met1 or Met1BC cells or 1.5 × 10^5^ 4T1 or 4T1 BC cells were prepared in 20 μl PBS and injected into the inguinal mammary fat pads of FVB/NH or BALB/cJ mice, respectively. Tumors were measured with calipers 2–3 times per week, and tumor volume was calculated as:

$$\left(3\right):{{{{{\rm{Volume}}}}}}=\frac{{{{{{{\rm{\pi }}}}}}{{{{{\rm{\times }}}}}}{{{{{\rm{long}}}}}}\,{{{{{\rm{axis}}}}}}\,\left({{{{{\rm{L}}}}}}\right){{{{{\rm{\times }}}}}}({{{{{\rm{short}}}}}}\,{{{{{\rm{axis}}}}}}\,({{{{{\rm{W}}}}}}))}^{2}}{6}$$

For spontaneous metastasis studies, 2.5 × 10^5^ Met1 BC Pool cells were prepared in 20 μl PBS and injected into two contralateral inguinal mammary fat pads of FVB mice. Mouse weight was monitored daily, and animals were sacrificed if their weight decreased by 20%. Otherwise, mice were sacrificed 21 days after injection by CO_2_ inhalation and perfused with PBS. Maximum permitted tumor burden is 1.5 cm; at no time was that tumor size exceeded. Tissues were collected for gDNA preparation and barcode analysis as described above.

### Cell proliferation assay

Met1 parental or barcoded clones were plated at a density of 20,000 cells/well in a 6-well plate in DMEM containing 10% fetal bovine serum, 100 U/mL penicillin-streptomycin, and 4 mM glutamine. Every 72 h over a 9-day time course, cells were counted using a hemocytometer and replated at a density of 20,000 cells/well. Relative proliferation rates of each BC are represented as cell doublings per day.

### Immunofluorescence and image analysis

Glass coverslips (#1.5; Election microscopy science) were coated with rat tail collagen I (Thermo-Fisher Scientific) overnight at room temperature. The coverslips were washed once with PBS, and cells were seeded and spread overnight at 37 °C and 5% CO_2_. The cells were then fixed in 4% paraformaldehyde (Sigma), permeabilized in 1% TX-100 (Sigma) and blocked in 3% bovine serum albumin in PBS (Fisher Scientific). Cells were stained with the indicated primary antibodies for overnight in 4 °C, followed by a 1-h incubation with secondary antibodies at room temperature. Then nuclei were visualized with DAPI (1 μg/ml, Sigma) and mounted on glass slides. Stains and primary antibodies: F-actin (rhodamine phalloidin, 1:1000; Thermo Fisher catalog: R415), CK14 (1:200; Biolegend catalog: 905303), and CK8 (1:10; TROMA-1, DSHB), anti-Zeb1 (1:100; Santa Cruz Biotechnology catalog: sc-25388). Secondary Alexa Fluor antibodies: anti-rat 647 (Invitrogen, 1:250, catalog: A21472), anti-rabbit 488 (Invitrogen, 1:250, catalog: A21206), anti-rat 488 (Invitrogen, 1:250, catalog: A21208), anti-rabbit 647 (Invitrogen, 1:250, catalog: A21244). Slides were imaged using a Nikon Eclipse Ni microscope. Numbers of CK8+ and CK14+ cells were counted by two independent researchers, represented as a percentage of total cells, and then stratified into 4 categories whereby 1 = 0–5%, 2 = 6–30%, 3 = 31–60% and 4 = 61–100% from 5 randomly selected images for each BC. Zeb1 quantification was performed using ImageJ by setting the threshold on the nuclear Zeb1 signal and on the DAPI (nucleus) stained image. The percentage of nuclear Zeb1+ cells was calculated as the number of nuclear Zeb1+ cells/field per total number of cells/field (DAPI).

### Flow cytometry

10^5^ cells were harvested using 0.25% trypsin, centrifuged, and seeded into round bottom 96-well plates. The cells were washed with FACS buffer (PBS with 2% FBS) and centrifuged at 300 × g for 5 min at 4 °C. Cells were incubated with anti-EpCAM (Clone G8.8, APC-Cy7, 1:400 dilution, BioLegend: 118218), anti-MHC-I (clone: KH114, FITC, 1:400 dilution, BioLegend: 115104), or anti-PD-L1 (clone: 10 F.9G2, PE, 1:100 dilution, BioLegend: 124308) on ice for 30 min. The cells were washed twice with FACS buffer and analyzed on a BD Canto II flow cytometer. DAPI (0.1 μg/ml, Sigma) was used as a dead cell marker. Data were analyzed using FlowJo software. Gating strategy is shown in Supplementary Fig. 7.

### Barcode association matrix

Each barcoded clone was assessed for 7 biological features via flow cytometry (EpCAM, MHC-I and PD-L1), Immunofluorescence (Zeb1, CK8, and CK14) and cell proliferation. Z-scores were generated for each quantified biological feature and assigned to each barcoded clone using the formula:

$$\left(4\right):Z=\frac{({{{{{{\rm{Value}}}}}}}_{{{{{{\rm{clone}}}}}}}{{{{{\rm{\hbox{-}}}}}}}{{{{{{\rm{Mean}}}}}}}_{{{{{{\rm{all}}}}}}{{{{{\rm{clones}}}}}}})}{{{{{{{\rm{Standard}}}}}}\,{{{{{\rm{Deviation}}}}}}}_{{{{{{\rm{all}}}}}}{{{{{\rm{clones}}}}}}}}$$

As an example, flow cytometry z-scores were calculated for each barcode using the mean fluorescence intensity (MFI) of each clone (Value_clone_), the average MFI of all clones (Mean_all clones_), and the standard deviation of the MFI for all clones (Standard Deviation_all clones_). A Pearson correlation matrix was generated by calculating the correlation of each barcode with every other barcode based on the 7 calculated z-scores. Similarly, a Pearson correlation matrix was generated by calculating the correlation of each biological feature with every other biological feature using the z-scores from the barcoded clones. Hierarchical clustering was performed using one minus Pearson’s correlation.

### Metastasis detection in the lung and bone

The total metastatic burden in various tissues was calculated by analyzing gDNA preparations as follows. The barcode region was pre-amplified from gDNA using the forward primer 5'-CGATTAGTGAACGGATCTCG-3' and the reverse primer 5'-CCGGTGGATGTGGAATGTG-3'. PCR mixtures contained 100 ng DNA, OneTaq 1x MasterMix (New England Biolabs), and 1 μM each of F/R primers. The following PCR program was used to amplify the template DNA: 94 °C for 30 s, followed by 15 cycles of 94 °C for 30 s, 52 °C for 30 s, and 68 °C for 30 s; a final extension was performed at 68 °C for 5 min. The PCR products were purified with a Monarch® PCR & DNA Cleanup Kit following manufacturer’s protocol. Purified PCR products were eluted in 17 μl water. Purified PCR products were used as input for qPCR to quantify all barcodes. For each 10-μl qPCR reaction, 4 μl of the purified PCR product was added to 1 μM primers and 2 μM probe (forward: 5'-TACCGGTTAGTAATGAC-3'; reverse: 5'-TAAAGCGCATGCTCCAG-3'; probe: 5'-FAM-AAAAGCGCCTCCCCTACCCGGTAGGTA-3'-Eclipse) and 5 μl TaqMan™ Fast Advanced Master Mix (Applied Biosystems™). The following PCR program was used: 50 °C for 2 min and 95 °C for 2 min, followed by 40 cycles of 95 °C for 3 s and 60 °C for 30 s.

### Calibration curve to estimate metastatic burden

To extrapolate the metastatic burden based on the total barcode qPCR signal obtained from different types of tissues, we first generated calibration curves by spiking known amounts of gDNA isolated from barcoded cells into gDNA isolated from a fixed amount of a given tissue from a tumor-free FVB mouse. Based on the estimated amount of gDNA (6 pg) per diploid cell^64^, we calculated the estimated barcoded cell number spiked into the gDNA amount equivalent to 0.1 g of indicated tissue. The serially diluted samples were then subjected to PCR-based barcode detection as described above (including pre-amplification, PCR product purification, and qPCR detection using primers and probes with TaqMan Fast Advanced Master Mix). Calibration curves were generated by linear regression analysis of the log-transformed estimated input of barcoded cells and log-transformed qPCR signals. The background signal was defined as the qPCR signal of the normal tissue without barcoded tumor cell gDNA. Samples with a signal higher than background were defined as positive for metastasis, and the metastatic burden was extrapolated from the calibration curve.

### Statistical analysis

The data are represented as the mean ± SEM, unless otherwise indicated. Data were analyzed by two-way ANOVA with the Sidak multiple comparison correction, one-way ANOVA with Tukey’s multiple comparison test for significance, or an unpaired two-tailed *t*-test with Welch’s correction, as indicated in the figure legends. All data were analyzed using GraphPad Prism 7.0. *P* < 0.05 was considered statistically significant. All data shown are representative of two or more independent experiments, unless otherwise indicated.

### Reporting summary

Further information on research design is available in the Nature Research Reporting Summary linked to this article.

## Supplementary information

Supplementary Information

Reporting Summary

## Data Availability

Source data are provided as a Source Data file. Barcode lentiviral vectors were generously provided by Dr. Todd R. Golub; barcode sequences are shown in Supplementary Fig. 1, as originally published in Additional Data File 2 of Peck, et al.^40^. Next generation sequencing was only used for barcode identification in this manuscript and the raw data are available upon request. Source data are provided with this paper.

## Source data

Source Data File

## Code availability

The scripts for identifying barcode sequences via NGS and for testing the distance between barcodes are available at https://github.com/petervangalen/BarcodeSimilarity, or the version used in this study 10.5281/zenodo.6585760^65^.
