# Supplementary material for: Clonal barcoding with qPCR detection enables live cell functional analyses for cancer research
Source: Nat Commun. 2022 Jul 4;13:3837. doi: 10.1038/s41467-022-31536-5 (PMC9252988; doi:10.1038/s41467-022-31536-5)
Supplement: Supplementary file 1 — Supplementary Information [file 41467_2022_31536_MOESM1_ESM.pdf]

Clonal Barcoding with qPCR Detection Enables Live Cell Functional Analyses for Cancer Research

Qiuchen Guo[1,2,*], Milos Spasic[1,2,*], Adam Maynard[2,*], Gregory J. Goreczny[1,2,*], Amanuel Bizuayehu[1], Jessica F. Olive[1,2], Peter van Galen[1,2,3], and Sandra S. McAllister[1-4]

[1]Division of Hematology, Department of Medicine, Brigham and Women's Hospital, Boston, MA 02115, USA
[2]Department of Medicine, Harvard Medical School, Boston, MA 02115, USA
[3]Broad Institute of Harvard and MIT, Cambridge, Massachusetts, 02142, USA
[4]Harvard Stem Cell Institute, Cambridge, Massachusetts, 02138, USA

Corresponding Author:
Sandra S. McAllister
Brigham & Women's Hospital
Harvard Institutes of Medicine 742
4 Blackfan Circle
Boston, MA 02115 USA
Phone: 617-525-4929
smcallister1@bwh.harvard.edu

* These authors contributed equally: Qiuchen Guo, Milos Spasic, Adam Maynard, Gregory J. Goreczny

SUPPLEMENTARY INFORMATION

SUPPLEMENTARY METHODS

Detection of Lung Metastases Using Bioluminescence Imaging

To analyze metastasis from orthotopic sites, 2.5×10^5 GFP-Luciferase-labeled parental Met1 I cells (GPF-Luc-Met1) were prepared in 20 µl sterile PBS and injected into the inguinal mammary fat pads. For intravenous injections, 7.5×10^5 GPF-Luc-Met1 cells were prepared in 100 µl sterile PBS and injected into the tail vein. 21 days after tumor cell injections, mice were euthanized, lungs were dissected and placed into wells, and covered with 300 µg/mL D-luciferin (Perkin-Elmer). Luciferase-positive cells were detected using a Xenogen IVIS imaging system (Caliper Life Sciences). Luminescent signal was detected as radiance (p/sec/cm2/sr) and analyzed using the Living Image Software Version 4.1 (Caliper Life Sciences).

SUPPLEMENTARY FIGURES

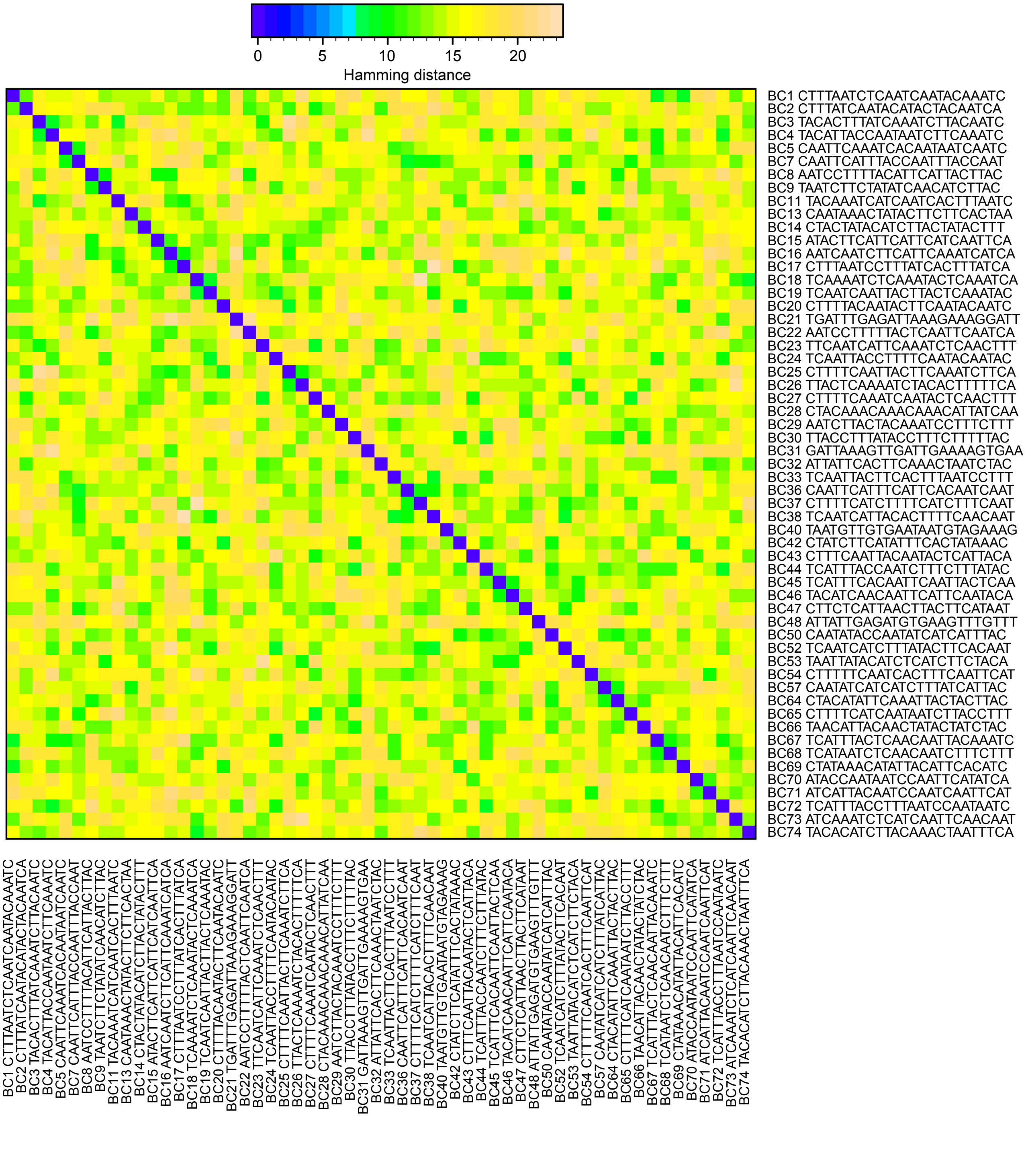

Supplementary Figure 1. Hamming Distances Between All Barcodes. Heatmap shows hamming distance between all barcode sequences used for SunCatcher. The minimum hamming distance between any 2 BCs is 9. The stringdist code is available at: https://github.com/petervangalen/BarcodeSimilarity

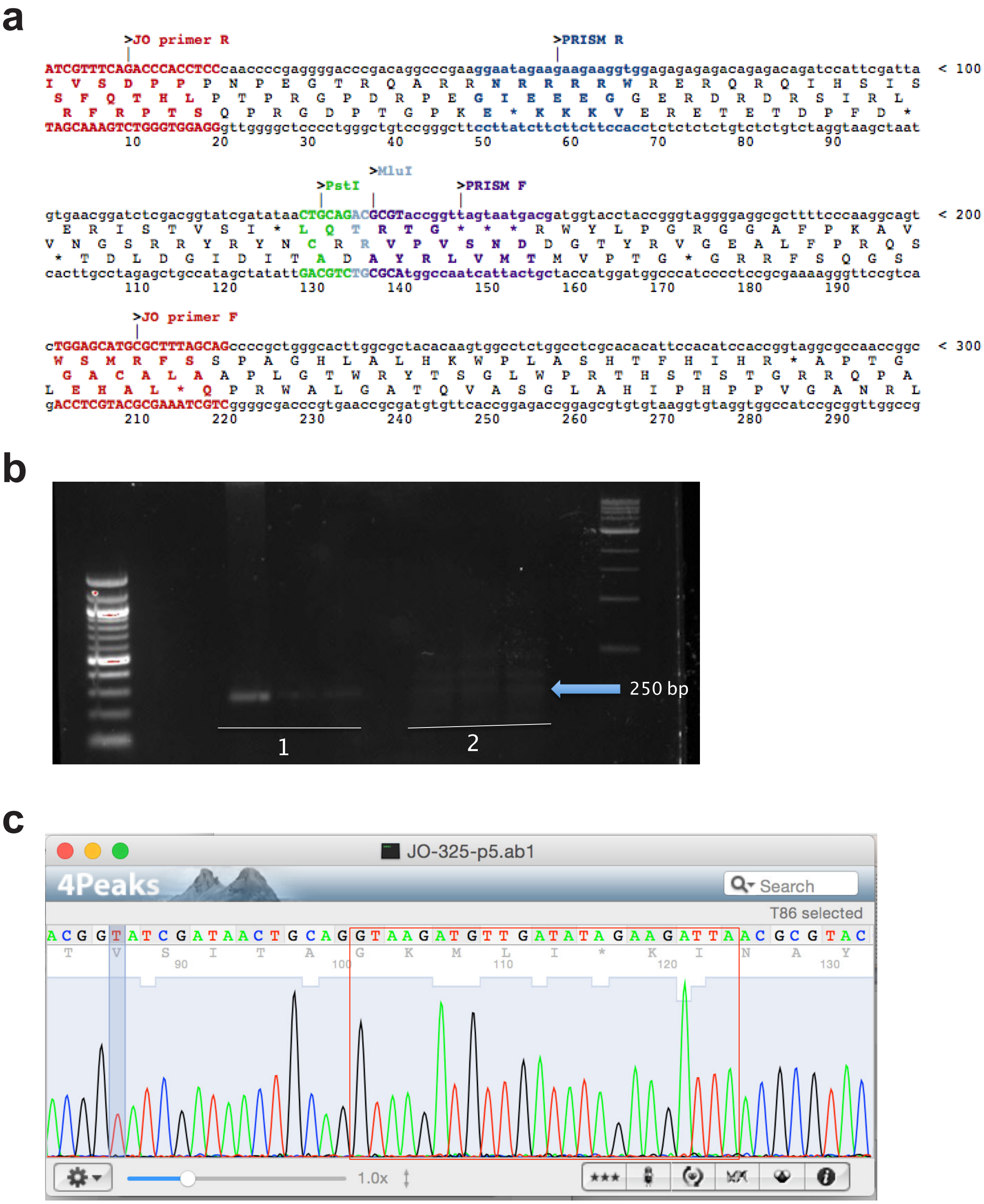

Supplementary Figure 2. Detection of Luminex DNA barcodes by Sanger sequencing. a Primers (JO primer F and JO primer R) were designed upstream and downstream of the barcode region (barcodes were inserted between PstI/MluI restriction sites) to use for PCR amplification prior to Sanger sequencing. **b** Agarose gel comparing PCR product yields from PCR reactions using gDNA input extracted using a Qiagen kit (designated as 1; lanes contained reactions with DNA input of 500 ng, 250 ng, and 100 ng, respectively) or the PRISM gDNA extraction method (designated as 2; lanes contained reactions with DNA input of 500 ng, 250 ng, and 100 ng, respectively). Expected amplicon size is ~250 bp (blue arrow). Micrograph is representative of 8 independent experiments. **c** Representative chromatogram from Sanger sequencing results of a Luminex barcode amplicon (barcode region is boxed in red).

a

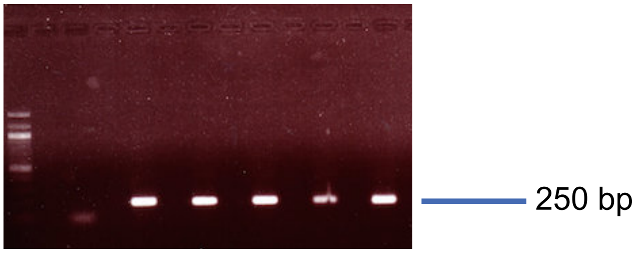

b

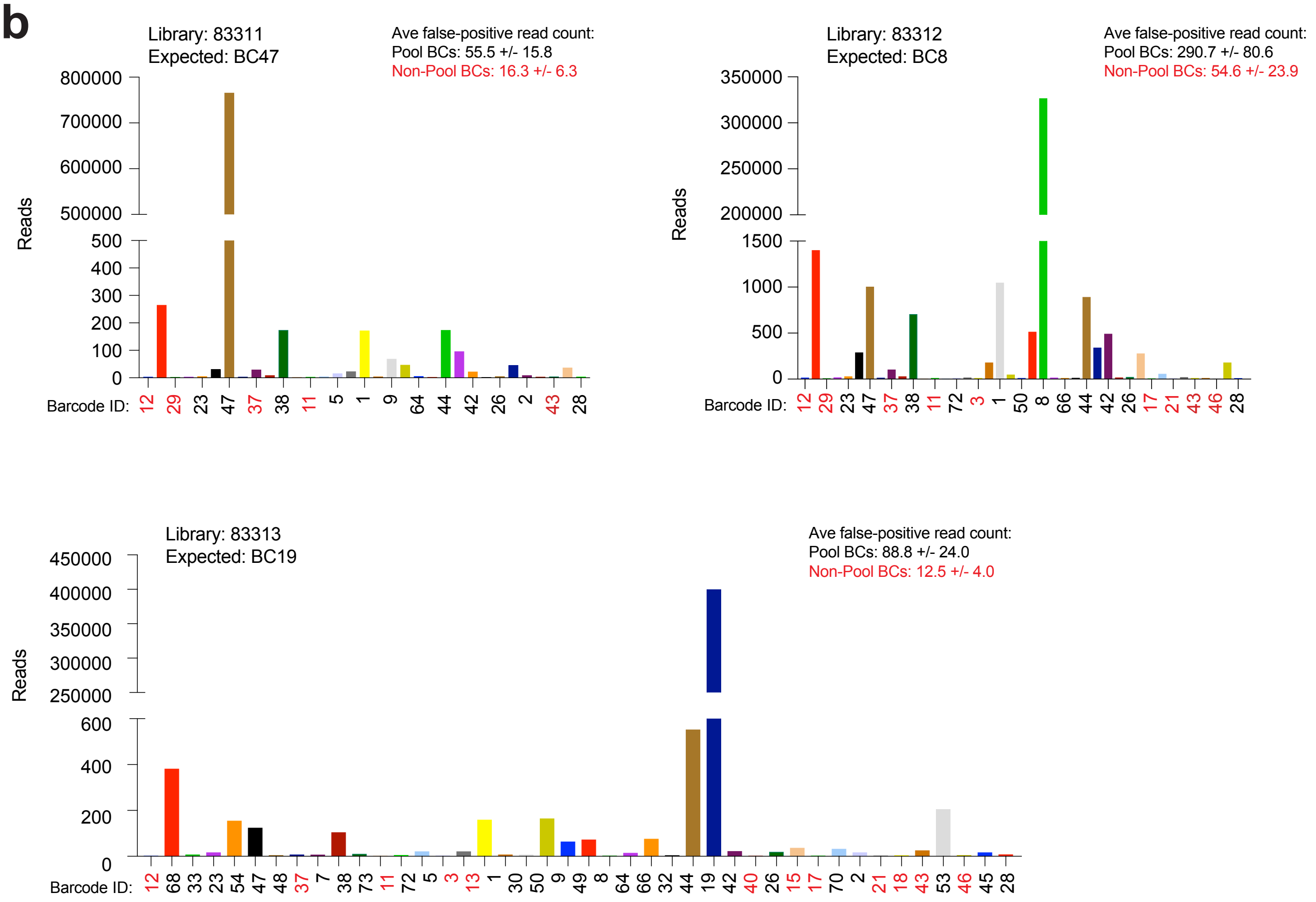

Supplementary Figure 3. Next Generation Sequencing Detection of BCs. a Agarose gel of PCR products following attachment of Illumina adaptors to barcode-index amplicons (Lane 1: 100 bp ladder; Lane 2: no DNA control; Lanes 3-7: PCR products). Micrograph is representative of 3 independent experiments. **b** Sequencing read counts from 3 HMLER-HR BC test samples using the Illumina library preparation method. Each library corresponds to a single Illumina adaptor, and the expected barcode pair for each library is indicated. For each library, the average false-positive read count (+/- S.E.M.) per BC is shown for BCs in the HMLER-HR BC Pool (black) and for BCs not represented in the HMLER-HR BC Pool (red). All BCs that yielded a read count are represented on the graphs. Source data are provided as a Source Data file.

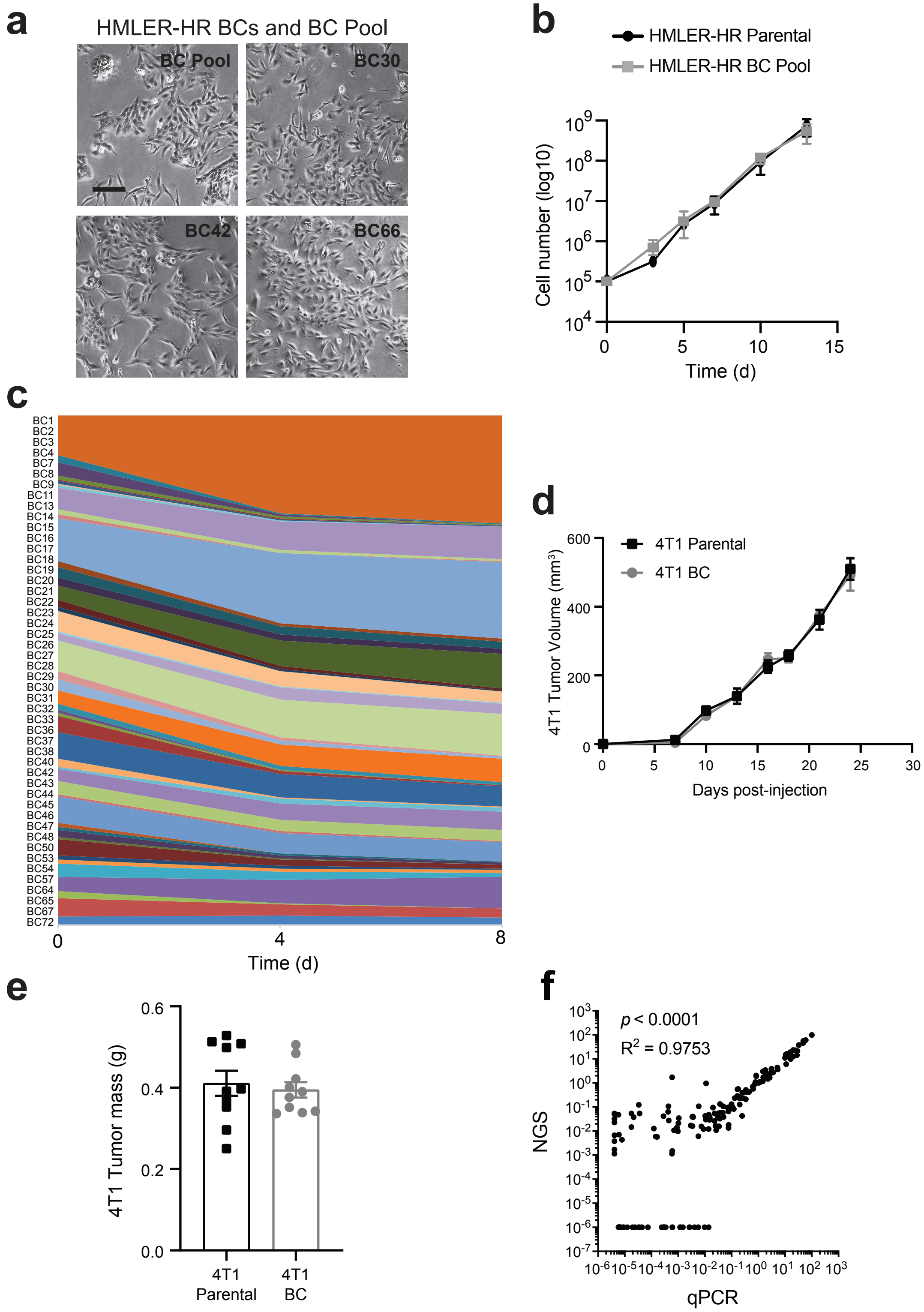

Supplementary Figure 4. Growth Properties of Barcoded Human and Mouse Cancer Cell Lines and Comparison of Deconvolution Methods. a Cell morphology of HMLER-HR barcode cells and the barcode pool. Scale bar = 200µm. Images are representative of 4 independent observations. **b** Growth kinetics of HMLER-HR Parental (black) and HMLER-HR BC Pool (grey) over 12 days in culture; n=3 replicates per group. Data are presented as mean values +/- SD. **c** Sand plot showing clonal composition (cumulative percentage) of 4T1 BC Pool over 8 days in vitro. **d** Growth of tumors from 4T1 parental cells (black) and 4T1 BC Pool cells (grey) in Balb/C mice (n=10 per cohort). Data are presented as mean values +/- SD **e** Mass (g) of 4T1 from (**d**) at 25-day experimental end point. Data are presented as mean values +/- SD **f** Genomic DNA was isolated from 6 tumors derived from the McNeu BC Pool after ~5 weeks of tumor growth in vivo; each barcode was quantified as a percentage of total barcodes in a given tumor by both qPCR and next-generation sequencing. Each data point represents the value for an individual barcode from an individual tumor by each method. Linear regression was used to calculate the data correlation between NGS and qPCR methods.Source data are provided as a Source Data file.

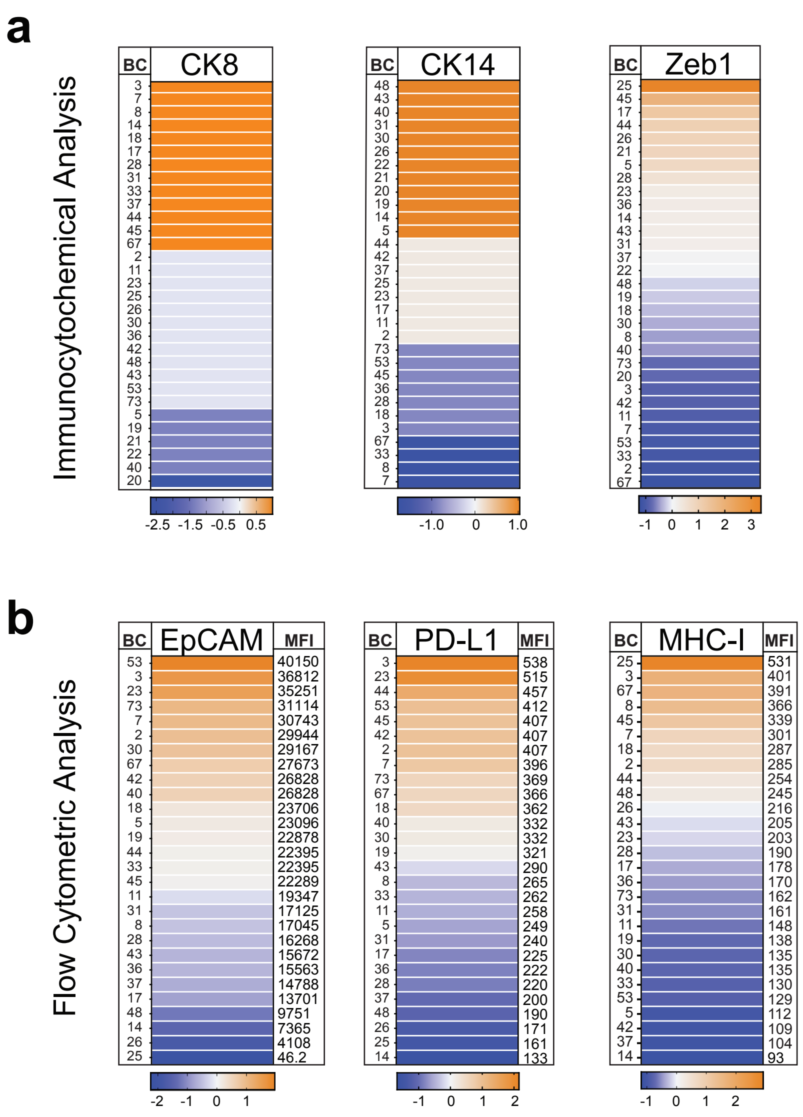

Supplementary Figure 5. BC Phenotype Analysis. a Heatmaps showing ranked relative expression levels of cytokeratin 8 (CK8), cytokeratin 14 (CK14), and nuclear Zeb 1 as quantified by immunofluorescence image analysis for each BC. **b** Heatmaps showing ranked relative expression of EpCAM, PD-L1, and MHC-I as quantified by flow cytometry for each BC. Median fluorescence intensity (MFI) is indicated for each BC. Data values provided in Supplementary Table 6. Flow cytometry gating strategy shown in Supplementary Figure 7.

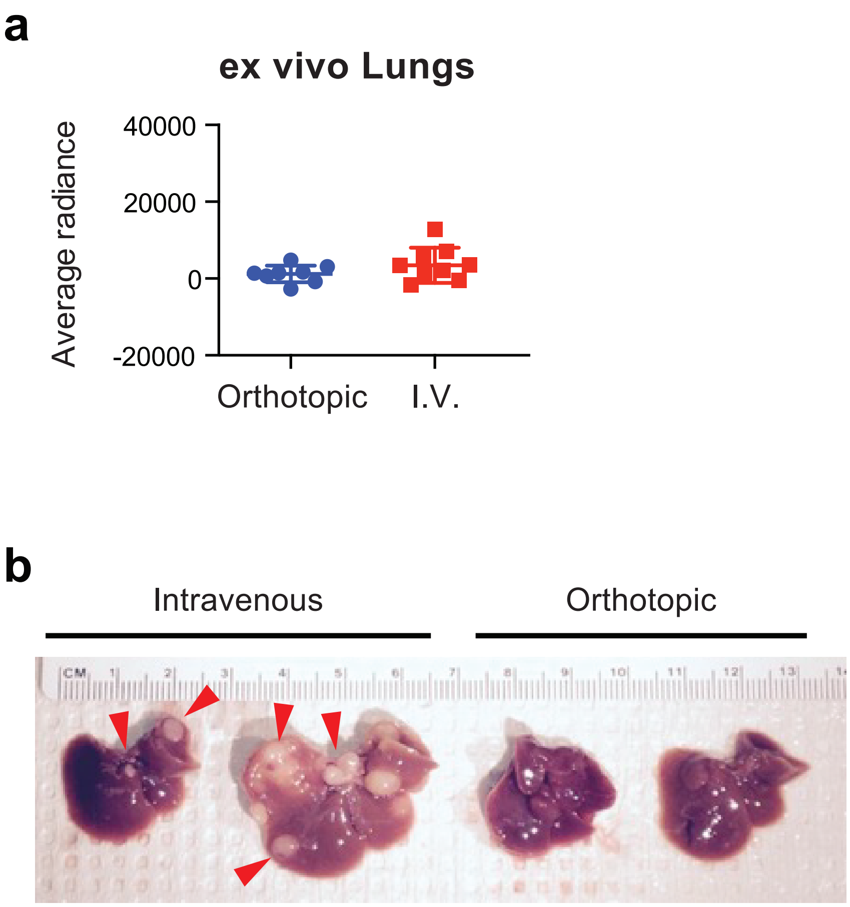

Supplementary Figure 6. Lung Metastases are not Detected After 21 Days Using Conventional Detection Method. **a** Average bioluminescent signal (radiance; p/sec/cm2/sr) in whole lung tissues 21 days after either orthotopic or intravenous (tail vein) injection of GFP-luciferase-tagged parental Met1 cells (n=9 per cohort). Error bars represent SEM. **b** Representative images of whole lungs from mice in (**a**). Red arrows indicate some examples of visible metastatic nodules.

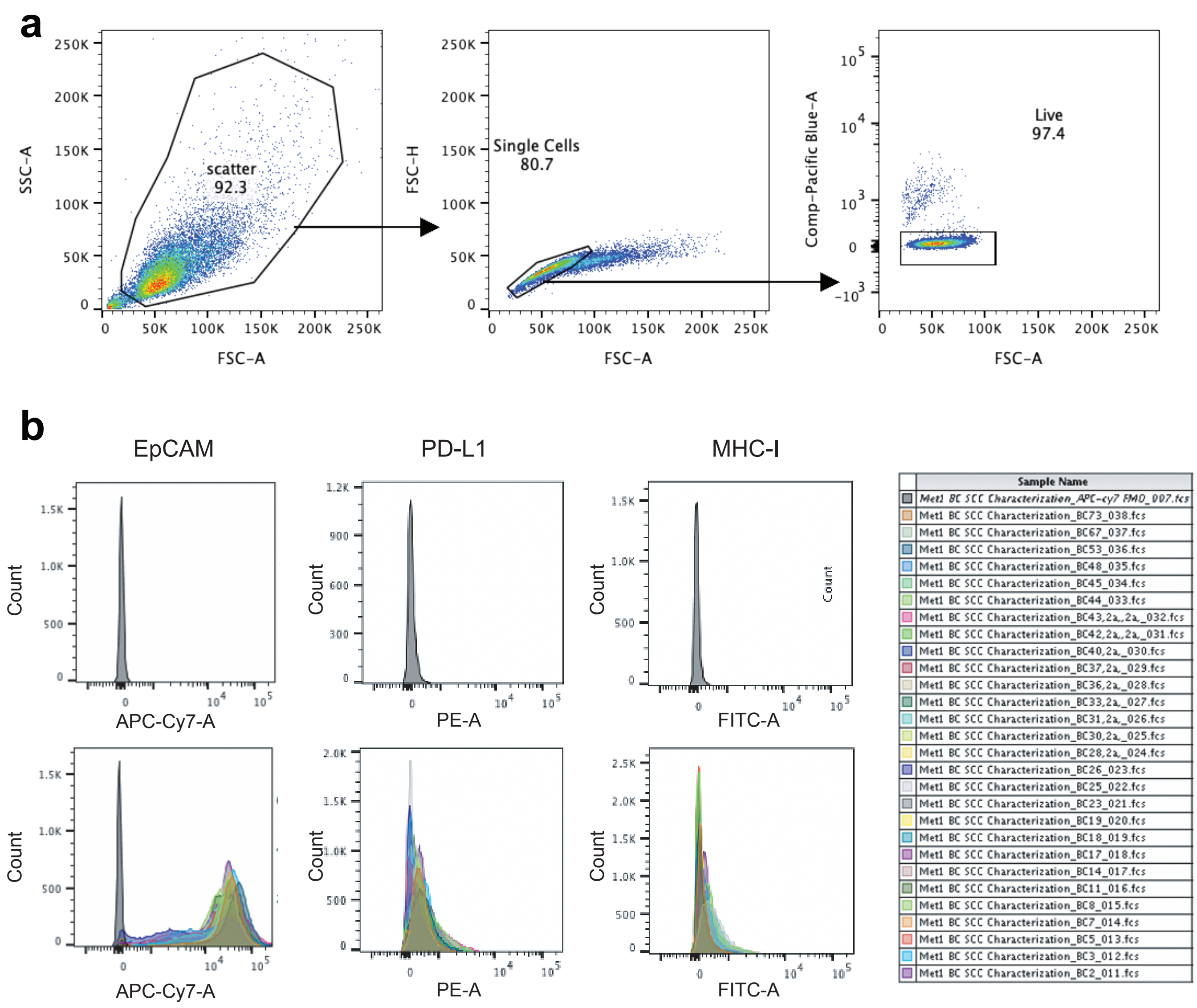

Supplementary Figure 7. Gating strategy and flow cytometric analysis. a Example of gating strategy used for single-color staining of Met1BCs. Cell debris is eliminated by FSC v SSC gating; single tumor cells are gated by FSC-A v FSC-H; live single cells are gated as DAPI-negative. Analysis of cell-surface markers is then performed on the live single cells. **b** Flow cytometric analysis of EpCAM, PD-L1, or MHC-I on indicated Met1 BCs. Histograms show unstained cells (top row) and staining results for all BCs (bottom row). Corresponds with data in Figure 4 and Supplementary Figure 5.

SUPPLEMENTARY TABLES

Supplementary Table 1. Collection of Clonally Barcoded Cell Lines

Supplementary Table 2. Oligonucleotide Primer Sequences for qPCR-based BC Detection

Supplementary Table 3. Barcode-index reverse (R) Primer Sequences for NGS-Based BC Detection.

Supplementary Table 4. Illumina Library Preparation Primer Sets.

Supplementary Table 5. Composition of Met1 BC Pool at Start and End of Injections. BC composition of the Met1 BC Pool was calculated at the initiation of injections ("pre-injection") and again approximately 2 hr later, when the final injection was performed ("post-injection"). The BC Pool cells were kept in suspension on ice during the injection time period.

Supplementary Table 6. Raw Data for Seven Biological Features of Individual Met1 BCs.

Supplementary Table 7. Z Scores for Seven Biological Features of Individual Met1 BCs (from data in Supplementary Table 6).

Supplementary Table 8. Pearson correlation Matrix (from data in Supplementary Table 7)

Supplementary Table 9. Multiple Variable Z Scores Used for Principal Component Analysis.

Supplementary Table 10. Multiple Variable Principal Component Analysis (from data in Supplementary Table 9).

Supplementary Table 11. Pearson Correlation Matrix of 7 Biological Features

Supplementary Table 1. Collection of clonally barcoded cell lines

BC Pool ID	Species	Breast Cancer Model	No. BCs	Subcloning Method (target dilution)	Estimated Frequency (%) of Wells with Single Viable Cell *	Barcode Identities
Met1 BC Pool	Murine	TNBC	31	Manual (0.5 cells/well)	32%	2 3 5 7 8 11 14 17 18 19 20 21 22 23 25 26 28 30 31 33 36 37 40 42 43 44 45 48 53 67 73
4T1 BC Pool	Murine	TNBC	48	Manual (0.5 cells/well)	25%	1 2 3 4 7 8 9 11 13 14 15 16 17 18 19 20 21 22 23 24 25 26 27 28 29 30 31 32 33 36 37 38 40 42 43 44 45 46 47 48 50 53 54 57 64 65 67 72
MeNeu BC Pool	Murine	Her2+	33	Manual (0.5 cells/well)	17%	1 2 3 4 5 7 8 9 11 12 13 15 17 18 21 23 24 26 29 30 31 32 33 37 38 40 42 43 44 45 46 47 48
HMLER-HR BC Pool	Human	TNBC	30	FACS (1 cell/well)	31%	1 2 5 7 8 9 19 23 26 28 30 32 33 38 42 44 45 47 48 49 50 53 54 64 66 67 68 70 72 73

*Parameters that impacted frequency:

No observable cell at time of plating or subsequent culture

More than one cell at time of plating

Clone not viable

Supplementary Table 2. Primer sequences for qPCR-based BC detection

Oligo ID	Sequence(5′-3′)	Length(bp)	GC content	Tm(°C)
BC1	GATTTGTATTGATTGAGATTAAAG	24	0.25	50.1
BC2	TGATTGTAGTATGTATTGATAAAG	24	0.25	50.1
BC3	GATTGTAAGATTTGATAAAGTGTA	24	0.25	50.1
BC4	GATTTGAAGATTATTGGTAATGTA	24	0.25	50.1
BC5	GATTGATTATTGTGATTTGAATTG	24	0.25	50.1
BC7	ATTGGTAAATTGGTAAATGAATTG	24	0.25	50.1
BC8	GTAAGTAATGAATGTAAAAGGATT	24	0.25	50.1
BC9	GTAAGATGTTGATATAGAAGATTA	24	0.25	50.1
BC11	GATTAAAGTGATTGATGATTTGTA	24	0.25	50.1
BC13	TTAGTGAAGAAGTATAGTTTATTG	24	0.25	50.1
BC14	AAAGTATAGTAAGATGTATAGTAG	24	0.25	50.1
BC15	TGAATTGATGAATGAATGAAGTAT	24	0.25	50.1
BC16	TGATGATTTGAATGAAGATTGATT	24	0.25	50.1
BC17	TGATAAAGTGATAAAGGATTAAAG	24	0.25	50.1
BC18	TGATTTGAGTATTTGAGATTTTGA	24	0.25	50.1
BC19	GTATTTGAGTAAGTAATTGATTGA	24	0.25	50.1
BC20	GATTGTATTGAAGTATTGTAAAAG	24	0.25	50.1
BC21	TGATTTGAGATTAAAGAAAGGATT	24	0.25	50.1
BC22	TGATTGAATTGAGTAAAAAGGATT	24	0.25	50.1
BC23	AAAGTTGAGATTTGAATGATTGAA	24	0.25	50.1
BC24	GTATTGTATTGAAAAGGTAATTGA	24	0.25	50.1
BC25	TGAAGATTTGAAGTAATTGAAAAG	24	0.25	50.1
BC26	TGAAAAAGTGTAGATTTTGAGTAA	24	0.25	50.1
BC27	AAAGTTGAGTATTGATTTGAAAAG	24	0.25	50.1
BC28	TTGATAATGTTTGTTTGTTTGTAG	24	0.25	50.1
BC29	AAAGAAAGGATTTGTAGTAAGATT	24	0.25	50.1
BC30	GTAAAAAGAAAGGTATAAAGGTAA	24	0.25	50.1
BC31	GATTAAAGTTGATTGAAAAGTGAA	24	0.25	50.1
BC32	GTAGATTAGTTTGAAGTGAATAAT	24	0.25	50.1
BC33	AAAGGATTAAAGTGAAGTAATTGA	24	0.25	50.1
BC36	ATTGATTGTGAATGAAATGAATTG	24	0.25	50.1
BC37	ATTGAAAGATGAAAAGATGAAAAG	24	0.25	50.1
BC38	ATTGTTGAAAAGTGTAATGATTGA	24	0.25	50.1
BC40	TAATGTTGTGAATAATGTAGAAAG	24	0.25	50.1
BC42	GTTTATAGTGAAATATGAAGATAG	24	0.25	50.1
BC43	TGTAATGAGTATTGTAATTGAAAG	24	0.25	50.1
BC44	GTATAAAGAAAGATTGGTAAATGA	24	0.25	50.1
BC45	TTGAGTAATTGAATTGTGAAATGA	24	0.25	50.1
BC46	TGTATTGAATGAATTGTTGATGTA	24	0.25	50.1
BC47	ATTATGAAGTAAGTTAATGAGAAG	24	0.25	50.1
BC48	ATTATTGAGATGTGAAGTTTGTTT	24	0.25	50.1
BC50	GTAAATGATGATATTGGTATATTG	24	0.25	50.1
BC52	ATTGTGAAGTATAAAGATGATTGA	24	0.25	50.1
BC53	TGTAGAAGATGAGATGTATAATTA	24	0.25	50.1
BC54	ATGAATTGAAAGTGATTGAAAAAG	24	0.25	50.1
BC57	GTAATGATAAAGATGATGATATTG	24	0.25	50.1
BC64	GTAAGTAGTAATTTGAATATGTAG	24	0.25	50.1
BC65	AAAGGTAAGATTATTGATGAAAAG	24	0.25	50.1
BC66	GTAGATAGTATAGTTGTAATGTTA	24	0.25	50.1
BC67	GATTTGTAATTGTTGAGTAAATGA	24	0.25	50.1
BC68	AAAGAAAGATTGTTGAGATTATGA	24	0.25	50.1
BC69	GATGTGAATGTAATATGTTTATAG	24	0.25	50.1
BC70	TGATATGAATTGGATTATTGGTAT	24	0.25	50.1
BC71	ATGAATTGATTGGATTGTAATGAT	24	0.25	50.1
BC72	GATTATTGGATTAAAGGTAAATGA	24	0.25	50.1
BC73	ATTGTTGAATTGATGAGATTTGAT	24	0.25	50.1
BC74	TGAAATTAGTTTGTAAGATGTGTA	24	0.25	50.1
PreAmp_F	CGATTAGTGAACGGATCTCG	20	0.5	60
PreAmp_R	CCGGTGGATGTGGAATGTG	19	0.58	60
BCUniversal	CCACTTGTGTAGCGCCAAG	19	0.58	60

Supplementary Table 3. Barcode-index reverse (R) primer sequences for NGS			
**Primer**	**Sequence (Reverse Complement)**	**Barcode-index #**	**Barcode Index Sequence**
JH p11	taggtgttcgtcattactaaccgg	1	aacaccta
JH p12	aggctggacgtcattactaaccgg	2	tccagcct
JH p13	gatgcaatcgtcattactaaccgg	3	attgcatc
JH p14	agctacgtcgtcattactaaccgg	4	acgtagct
JH p31	cagttagtcgtcattactaaccgg	5	actaactg
JH p32	atcttaagcgtcattactaaccgg	6	cttaagat
JH p33	ctgtttaacgtcattactaaccgg	7	ttaaacag
JH p34	cgaattcacgtcattactaaccgg	8	tgaattcg
JH p35	aataggatcgtcattactaaccgg	9	atcctatt
JH p36	tcctatatcgtcattactaaccgg	10	atatagga
JH p37	tagcaactcgtcattactaaccgg	11	agttgcta
JH p38	actagctacgtcattactaaccgg	12	tagctagt
JH p39	gttaactccgtcattactaaccgg	13	gagttaac
JH p40	gaatctagcgtcattactaaccgg	14	ctagattc
JH p41	gattcgtacgtcattactaaccgg	15	tacgaatc
JH p42	gcaatcttcgtcattactaaccgg	16	aagattgc
JH p43	acggtatacgtcattactaaccgg	17	tataccgt
JH p44	tgtgactacgtcattactaaccgg	18	tagtcaca
JH p45	tcagcattcgtcattactaaccgg	19	aatgctga
JH p46	ataacggtcgtcattactaaccgg	20	accgttat
PreAmp F (5'-3')	TGGAGCATGCGCTTTAGCAG		
PreAmp R (5'-3')	ATCGTTTCAGACCCACCTCC		

Supplementary Table 4. Illumina Library Preparation Primer Sets for NGS

Primer	TruSeq Index	Primer Sequence
JO p50	n/a	AATGATACGGCGACCACCGAGATCTACACTCTTTCCCTACACGACGCTCTTCCGATCTgacggtatcgatataaCTGCAG
JO p65	(1) CGTGAT	CAAGCAGAAGACGGCATACGAGATCGTGATGTGACTGGAGTTCAGACGTGTGCTCTTCCGATCTcgtcattactaaccgg
JO p66	(2) ACATCG	CAAGCAGAAGACGGCATACGAGATACATCGGTGACTGGAGTTCAGACGTGTGCTCTTCCGATCTcgtcattactaaccgg
JO p67	(3) GCCTAA	CAAGCAGAAGACGGCATACGAGATGCCTAAGTGACTGGAGTTCAGACGTGTGCTCTTCCGATCTcgtcattactaaccgg
JO p68	(4) TGGTCA	CAAGCAGAAGACGGCATACGAGATTGGTCAGTGACTGGAGTTCAGACGTGTGCTCTTCCGATCTcgtcattactaaccgg
JO p69	(5) CACTGT	CAAGCAGAAGACGGCATACGAGATCACTGTGTGACTGGAGTTCAGACGTGTGCTCTTCCGATCTcgtcattactaaccgg
JO p70	(6) ATTGGC	CAAGCAGAAGACGGCATACGAGATATTGGCGTGACTGGAGTTCAGACGTGTGCTCTTCCGATCTcgtcattactaaccgg
JO p71	(7) GATCTG	CAAGCAGAAGACGGCATACGAGATGATCTGGTGACTGGAGTTCAGACGTGTGCTCTTCCGATCTcgtcattactaaccgg
JO p72	(8) TCAAGT	CAAGCAGAAGACGGCATACGAGATTCAAGTGTGACTGGAGTTCAGACGTGTGCTCTTCCGATCTcgtcattactaaccgg
JO p73	(9) CTGATC	CAAGCAGAAGACGGCATACGAGATCTGATCGTGACTGGAGTTCAGACGTGTGCTCTTCCGATCTcgtcattactaaccgg
JO p74	(10) AAGCTA	CAAGCAGAAGACGGCATACGAGATAAGCTAGTGACTGGAGTTCAGACGTGTGCTCTTCCGATCTcgtcattactaaccgg
JO p75	(11) GTAGCC	CAAGCAGAAGACGGCATACGAGATGTAGCCGTGACTGGAGTTCAGACGTGTGCTCTTCCGATCTcgtcattactaaccgg
JO p76	(12) TACAAG	CAAGCAGAAGACGGCATACGAGATTACAAGGTGACTGGAGTTCAGACGTGTGCTCTTCCGATCTcgtcattactaaccgg
JO p77	(13) TTGACT	CAAGCAGAAGACGGCATACGAGATTTGACTGTGACTGGAGTTCAGACGTGTGCTCTTCCGATCTcgtcattactaaccgg
JO p78	(14) GGAACT	CAAGCAGAAGACGGCATACGAGATGGAACTGTGACTGGAGTTCAGACGTGTGCTCTTCCGATCTcgtcattactaaccgg
JO p79	(15) TGACAT	CAAGCAGAAGACGGCATACGAGATTGACATGTGACTGGAGTTCAGACGTGTGCTCTTCCGATCTcgtcattactaaccgg
JO p80	(16) GGACGG	CAAGCAGAAGACGGCATACGAGATGGACGGGTGACTGGAGTTCAGACGTGTGCTCTTCCGATCTcgtcattactaaccgg
JO p81	(18) GCGGAC	CAAGCAGAAGACGGCATACGAGATGCGGACGTGACTGGAGTTCAGACGTGTGCTCTTCCGATCTcgtcattactaaccgg
JO p82	(19) TTTCAC	CAAGCAGAAGACGGCATACGAGATTTTCACGTGACTGGAGTTCAGACGTGTGCTCTTCCGATCTcgtcattactaaccgg
JO p83	(20) GGCCAC	CAAGCAGAAGACGGCATACGAGATGGCCACGTGACTGGAGTTCAGACGTGTGCTCTTCCGATCTcgtcattactaaccgg
JO p84	(21) CGAAAC	CAAGCAGAAGACGGCATACGAGATCGAAACGTGACTGGAGTTCAGACGTGTGCTCTTCCGATCTcgtcattactaaccgg
JO p85	(22) CGTACG	CAAGCAGAAGACGGCATACGAGATCGTACGGTGACTGGAGTTCAGACGTGTGCTCTTCCGATCTcgtcattactaaccgg
JO p86	(23) CCACTC	CAAGCAGAAGACGGCATACGAGATCCACTCGTGACTGGAGTTCAGACGTGTGCTCTTCCGATCTcgtcattactaaccgg
JO p87	(25) ATCAGT	CAAGCAGAAGACGGCATACGAGATATCAGTGTGACTGGAGTTCAGACGTGTGCTCTTCCGATCTcgtcattactaaccgg
JO p88	(27) AGGAAT	CAAGCAGAAGACGGCATACGAGATAGGAATGTGACTGGAGTTCAGACGTGTGCTCTTCCGATCTcgtcattactaaccgg

Supplementary Table 5. Composition of Met1 BC Pool At First and Last Injections

	% Composition	
BC	Pre-Injection	Post-Injection
2	0.746	0.820
3	0.222	0.170
5	0.109	0.118
7	4.351	4.749
8	2.057	2.657
11	1.570	1.597
14	0.240	0.244
17	0.021	0.020
18	1.035	1.172
19	2.876	2.972
20	8.315	9.679
21	0.456	0.544
22	1.224	1.501
23	1.776	1.809
25	0.788	0.530
26	0.672	0.687
28	0.391	0.283
30	1.094	1.100
31	1.046	0.974
33	0.340	0.305
36	2.729	2.297
37	0.059	0.070
40	0.277	0.285
42	1.354	1.369
43	4.118	5.605
44	0.889	0.883
45	13.140	11.098
48	0.166	0.142
53	25.619	24.696
67	20.512	19.926
73	1.808	1.696

Supplementary Table 6. Raw data for seven biological features of individual Met1 BCs

	Flow Cytometry Mean Fluorescence Intensity			Immunofluorescence Analysis			Cell count
**Clone**	**MHC-I (MFI)**	**EpCAM (MFI)**	**PD-L1 (MFI)**	**Zeb1 (% nuclei)**	**CK8 (score)**	**CK14 (score)**	**proliferation slope**
2	285	29944	407	10.44	3	3	1.01
3	401	36812	538	14.45	4	2	0.84
5	112	23096	249	43.13	2	4	1.09
7	301	30743	396	12.41	4	1	0.98
8	366	17045	265	21.63	4	1	1.00
11	148	19347	258	13.57	3	3	1.01
14	92.5	7365	133	33.64	4	4	1.08
17	178	13701	225	52.11	4	3	0.81
18	287	23706	362	24.42	4	2	1.23
19	138	22878	321	25.83	2	4	1.22
20	NA	NA	NA	15.89	1	4	0.64
21	NA	NA	NA	44.97	2	4	1.18
22	NA	NA	NA	29.99	2	4	1.09
23	203	35251	515	34.37	3	3	0.90
25	531	46.2	161	88.11	3	3	1.45
26	216	4108	171	46.01	3	4	0.94
28	190	16268	220	38.76	4	2	0.84
30	135	29167	332	22.99	3	4	1.08
31	161	17125	240	32.99	4	4	0.89
33	130	22395	262	11.55	4	1	1.12
36	170	15563	222	33.98	3	2	1.00
37	104	14788	200	30.34	4	3	1.13
40	135	26828	332	21.08	2	4	1.08
42	109	26828	407	14.18	3	3	1.16
43	205	15672	290	33.60	3	4	1.18
44	254	22395	457	47.70	4	3	1.14
45	339	22289	407	62.48	4	2	1.14
48	245	9751	190	26.53	3	4	0.90
53	129	40150	412	11.94	3	2	1.23
67	391	27673	366	7.89	4	1	1.24
73	162	31114	369	15.91	3	2	1.25

Supplementary Table 7. Z scores for seven biological features of individual Met1 BCs (from data in Supplementary Table 6)

Clone	MHC-I	EpCAM	PD-L1	nuclear Zeb1	CK8	CK14	proliferation
2	0.622	0.888	0.912	-1.116	-0.236	0.092	-0.288
3	1.707	1.610	2.156	-0.885	0.984	-0.854	-1.345
5	-0.996	0.168	-0.588	0.772	-1.456	1.037	0.192
7	0.772	0.972	0.808	-1.003	0.984	-1.799	-0.492
8	1.380	-0.469	-0.437	-0.470	0.984	-1.799	-0.373
11	-0.659	-0.227	-0.503	-0.936	-0.236	0.092	-0.330
14	-1.178	-1.487	-1.690	0.223	0.984	1.037	0.146
17	-0.379	-0.820	-0.816	1.290	0.984	0.092	-1.564
18	0.641	0.232	0.485	-0.309	0.984	-0.854	1.070
19	-0.753	0.145	0.095	-0.228	-1.456	1.037	0.975
23	-0.145	1.446	1.938	0.265	-0.236	0.092	-0.967
25	2.923	-2.256	-1.424	3.369	-0.236	0.092	2.389
26	-0.023	-1.829	-1.329	0.938	-0.236	1.037	-0.737
28	-0.266	-0.550	-0.864	0.519	0.984	-0.854	-1.344
30	-0.781	0.806	0.200	-0.392	-0.236	1.037	0.116
31	-0.538	-0.460	-0.674	0.186	0.984	1.037	-1.071
33	-0.828	0.094	-0.465	-1.052	0.984	-1.799	0.386
36	-0.453	-0.625	-0.845	0.243	-0.236	-0.854	-0.373
37	-1.071	-0.706	-1.054	0.033	0.984	0.092	0.437
40	-0.781	0.560	0.200	-0.502	-1.456	1.037	0.119
42	-1.024	0.560	0.912	-0.900	-0.236	0.092	0.618
43	-0.126	-0.613	-0.199	0.221	-0.236	1.037	0.733
44	0.332	0.094	1.387	1.035	0.984	0.092	0.506
45	1.127	0.083	0.912	1.889	0.984	-0.854	0.513
48	0.248	-1.236	-1.149	-0.187	-0.236	1.037	-0.969
53	-0.837	1.961	0.959	-1.029	-0.236	-0.854	1.036
67	1.613	0.649	0.523	-1.263	0.984	-1.799	1.119
73	-0.528	1.011	0.551	-0.800	-0.236	-0.854	1.172

Supplementary Table 8. Pearson Correlation Matrix (from data in Supplementary Table 7)

BC	73	42	53	23	2	11	30	67	18	8	7	3	33	48	43	40	19	5	45	44	36	28	25	37	14	31	17	26
73	1.000	0.779	0.953	0.218	0.412	0.180	0.278	0.552	0.576	-0.027	0.438	0.185	0.618	-0.873	-0.282	0.258	0.303	-0.111	-0.142	-0.024	-0.286	-0.538	-0.418	-0.123	-0.480	-0.716	-0.795	-0.888
42	0.779	1.000	0.820	0.423	0.469	0.527	0.681	0.105	0.170	-0.474	0.135	0.115	0.296	-0.601	0.002	0.585	0.538	0.106	-0.501	0.052	-0.641	-0.646	-0.701	-0.067	-0.268	-0.341	-0.676	-0.683
53	0.953	0.820	1.000	0.447	0.537	0.266	0.442	0.435	0.405	-0.124	0.482	0.324	0.542	-0.861	-0.414	0.362	0.276	-0.064	-0.243	-0.070	-0.393	-0.478	-0.639	-0.219	-0.549	-0.589	-0.713	-0.934
23	0.218	0.423	0.447	1.000	0.604	-0.057	0.342	-0.093	-0.240	-0.231	0.379	0.662	-0.136	-0.430	-0.574	0.346	0.000	0.000	-0.033	0.268	-0.429	-0.098	-0.705	-0.654	-0.686	-0.151	-0.107	-0.514
2	0.412	0.469	0.537	0.604	1.000	0.325	0.303	0.442	0.159	0.091	0.581	0.786	0.035	-0.283	-0.467	0.334	0.046	-0.337	-0.398	-0.232	-0.823	-0.449	-0.658	-0.712	-0.735	-0.360	-0.605	-0.680
11	0.180	0.527	0.266	-0.057	0.325	1.000	0.779	-0.127	-0.139	-0.422	-0.148	-0.091	0.078	0.207	0.299	0.486	0.376	0.119	-0.922	-0.518	-0.675	-0.344	-0.594	0.308	0.339	0.376	-0.269	-0.119
30	0.278	0.681	0.442	0.342	0.303	0.779	1.000	-0.461	-0.491	-0.823	-0.330	-0.157	-0.197	-0.019	0.267	0.854	0.681	0.567	-0.866	-0.439	-0.637	-0.508	-0.674	0.031	0.082	0.191	-0.306	-0.193
67	0.552	0.105	0.435	-0.093	0.442	-0.127	-0.461	1.000	0.921	0.786	0.824	0.548	0.709	-0.517	-0.550	-0.475	-0.428	-0.795	0.273	0.058	-0.061	-0.043	-0.043	-0.191	-0.478	-0.580	-0.483	-0.657
18	0.576	0.170	0.405	-0.240	0.159	-0.139	-0.491	0.921	1.000	0.724	0.704	0.320	0.854	-0.571	-0.402	-0.576	-0.414	-0.768	0.371	0.281	0.117	0.042	0.033	0.122	-0.231	-0.497	-0.377	-0.571
8	-0.027	-0.474	-0.124	-0.231	0.091	-0.422	-0.823	0.786	0.724	1.000	0.722	0.503	0.522	-0.123	-0.577	-0.869	-0.837	-0.871	0.605	0.214	0.343	0.503	0.261	-0.056	-0.216	-0.173	0.122	-0.179
7	0.438	0.135	0.482	0.379	0.581	-0.148	-0.330	0.824	0.704	0.722	1.000	0.865	0.663	-0.585	-0.900	-0.473	-0.646	-0.837	0.320	0.242	-0.111	0.231	-0.414	-0.333	-0.619	-0.346	-0.205	-0.749
3	0.185	0.115	0.324	0.662	0.786	-0.091	-0.157	0.548	0.320	0.503	0.865	1.000	0.235	-0.343	-0.843	-0.220	-0.534	-0.686	0.146	0.195	-0.388	0.136	-0.544	-0.632	-0.731	-0.205	-0.143	-0.608
33	0.618	0.296	0.542	-0.136	0.035	0.078	-0.197	0.709	0.854	0.522	0.663	0.235	1.000	-0.642	-0.481	-0.506	-0.427	-0.625	0.233	0.242	0.189	0.235	-0.250	0.395	-0.032	-0.212	-0.173	-0.612
48	-0.873	-0.601	-0.861	-0.430	-0.283	0.207	-0.019	-0.517	-0.571	-0.123	-0.585	-0.343	-0.642	1.000	0.558	-0.003	-0.005	0.218	-0.273	-0.345	-0.001	0.207	0.371	0.203	0.599	0.693	0.500	0.871
43	-0.282	0.002	-0.414	-0.574	-0.467	0.299	0.267	-0.550	-0.402	-0.577	-0.900	-0.843	-0.481	0.558	1.000	0.405	0.667	0.610	-0.406	-0.241	-0.055	-0.403	0.435	0.406	0.647	0.221	-0.040	0.644
40	0.258	0.585	0.362	0.346	0.334	0.486	0.854	-0.475	-0.576	-0.869	-0.473	-0.220	-0.506	-0.003	0.405	1.000	0.899	0.742	-0.746	-0.466	-0.636	-0.782	-0.375	-0.306	-0.159	-0.145	-0.503	-0.122
19	0.303	0.538	0.276	0.000	0.046	0.376	0.681	-0.428	-0.414	-0.837	-0.646	-0.534	-0.427	-0.005	0.667	0.899	1.000	0.791	-0.616	-0.393	-0.425	-0.852	-0.045	-0.083	0.031	-0.266	-0.562	0.009
5	-0.111	0.106	-0.064	0.000	-0.337	0.119	0.567	-0.795	-0.768	-0.871	-0.837	-0.686	-0.625	0.218	0.610	0.742	0.791	1.000	-0.380	-0.364	0.013	-0.393	0.135	0.042	0.238	0.080	-0.013	0.374
45	-0.142	-0.501	-0.243	-0.033	-0.398	-0.922	-0.866	0.273	0.371	0.605	0.320	0.146	0.233	-0.273	-0.406	-0.746	-0.616	-0.380	1.000	0.682	0.760	0.565	0.507	-0.048	-0.183	-0.235	0.389	0.060
44	-0.024	0.052	-0.070	0.268	-0.232	-0.518	-0.439	0.058	0.281	0.214	0.242	0.195	0.242	-0.345	-0.241	-0.466	-0.393	-0.364	0.682	1.000	0.309	0.363	0.027	0.067	-0.075	-0.041	0.304	-0.056
36	-0.286	-0.641	-0.393	-0.429	-0.823	-0.675	-0.637	-0.061	0.117	0.343	-0.111	-0.388	0.189	-0.001	-0.055	-0.636	-0.425	0.013	0.760	0.309	1.000	0.660	0.662	0.429	0.335	0.098	0.598	0.391
28	-0.538	-0.646	-0.478	-0.098	-0.449	-0.344	-0.508	-0.043	0.042	0.503	0.231	0.136	0.235	0.207	-0.403	-0.782	-0.852	-0.393	0.565	0.363	0.660	1.000	0.143	0.378	0.337	0.587	0.884	0.333
25	-0.418	-0.701	-0.639	-0.705	-0.658	-0.594	-0.674	-0.043	0.033	0.261	-0.414	-0.544	-0.250	0.371	0.435	-0.375	-0.045	0.135	0.507	0.027	0.662	0.143	1.000	0.172	0.302	-0.115	0.229	0.629
37	-0.123	-0.067	-0.219	-0.654	-0.712	0.308	0.031	-0.191	0.122	-0.056	-0.333	-0.632	0.395	0.203	0.406	-0.306	-0.083	0.042	-0.048	0.067	0.429	0.378	0.172	1.000	0.895	0.541	0.404	0.372
14	-0.480	-0.268	-0.549	-0.686	-0.735	0.339	0.082	-0.478	-0.231	-0.216	-0.619	-0.731	-0.032	0.599	0.647	-0.159	0.031	0.238	-0.183	-0.075	0.335	0.337	0.302	0.895	1.000	0.735	0.536	0.712
31	-0.716	-0.341	-0.589	-0.151	-0.360	0.376	0.191	-0.580	-0.497	-0.173	-0.346	-0.205	-0.212	0.693	0.221	-0.145	-0.266	0.080	-0.235	-0.041	0.098	0.587	-0.115	0.541	0.735	1.000	0.783	0.630
17	-0.795	-0.676	-0.713	-0.107	-0.605	-0.269	-0.306	-0.483	-0.377	0.122	-0.205	-0.143	-0.173	0.500	-0.040	-0.503	-0.562	-0.013	0.389	0.304	0.598	0.884	0.229	0.404	0.536	0.783	1.000	0.666
26	-0.888	-0.683	-0.934	-0.514	-0.680	-0.119	-0.193	-0.657	-0.571	-0.179	-0.749	-0.608	-0.612	0.871	0.644	-0.122	0.009	0.374	0.060	-0.056	0.391	0.333	0.629	0.372	0.712	0.630	0.666	1.000

Supplementary Table 9. Multiple Variable Z scores used for principal component analysis (from data in Table 1 and Supplementary Table 7)

ID Number	Tumor Composition	Proliferation	in vitro FC	In vivo FC	MHC-I	EpCAM	PD-L1	Zeb1	CK8	CK14
2	>1%	-0.2882	0.227981	0.234441	0.6221	0.8877	0.912	-1.1162	-0.2361	0.0915
7	>1%	-0.4921	0.421743	0.232579	0.7717	0.9717	0.8075	-1.0027	0.9836	-1.7986
25	>1%	2.389	0.235917	0.234963	2.9226	-2.2561	-1.4241	3.3686	-0.2361	0.0915
8	>1%	-0.373	0.322177	0.236249	1.3795	-0.4686	-0.4365	-0.4698	0.9836	-1.7986
53	>1%	1.0359	0.646465	0.23283	-0.8368	1.9609	0.9594	-1.0294	-0.2361	-0.8536
67	>1%	1.1188	0.592505	0.234242	1.6133	0.6489	0.5226	-1.2632	0.9836	-1.7986
3	0.01-.99%	-1.3449	-0.121281	0.233746	1.7069	1.6099	2.156	-0.8848	0.9836	-0.8536
18	0.01-.99%	1.0701	0.269289	0.079022	0.6408	0.2318	0.4846	-0.3088	0.9836	-0.8536
19	0.01-.99%	0.9748	0.337433	0.22607	-0.7526	0.1447	0.0953	-0.2277	-1.4557	1.0365
20	0.01-.99%	-2.5958	0.463628	0.201196	NA	NA	NA	-0.8014	-2.6754	1.0365
22	0.01-.99%	0.2014	0.285724	0.203104	NA	NA	NA	0.0126	-1.4557	1.0365
23	0.01-.99%	-0.9674	0.313685	0.222026	-0.1448	1.4457	1.9375	0.2654	-0.2361	0.0915
31	0.01-.99%	-1.0707	0.270418	0.232494	-0.5376	-0.4602	-0.6739	0.1861	0.9836	1.0365
33	0.01-.99%	0.3859	0.051501	0.22752	-0.8275	0.0939	-0.465	-1.0521	0.9836	-1.7986
36	0.01-.99%	-0.373	0.335373	0.215318	-0.4534	-0.6245	-0.8448	0.2432	-0.2361	-0.8536
40	0.01-.99%	0.1188	-0.022379	0.229717	-0.7807	0.5601	0.1998	-0.5017	-1.4557	1.0365
42	0.01-.99%	0.6181	0.294334	0.205568	-1.0238	0.5601	0.912	-0.9002	-0.2361	0.0915
43	0.01-.99%	0.7332	0.419284	0.226913	-0.1261	-0.613	-0.1991	0.2211	-0.2361	1.0365
45	0.01-.99%	0.5133	0.514616	0.17489	1.127	0.0828	0.912	1.8885	0.9836	-0.8536
73	<0.01%	1.1716	0.314793	-5.357475	-0.5282	1.0107	0.5511	-0.8002	-0.2361	-0.8536
21	<0.01%	0.7202	0.134044	-0.256057	NA	NA	NA	0.8774	-1.4557	1.0365
37	<0.01%	0.4373	-1.502868	0.225176	-1.0706	-0.706	-1.0537	0.0329	0.9836	0.0915
44	<0.01%	0.5058	0.251798	0.193677	0.3322	0.0939	1.3868	1.0353	0.9836	0.0915
5	<0.01%	0.192	-0.636394	0.228388	-0.9958	0.1676	-0.5884	0.7716	-1.4557	1.0365
14	<0.01%	0.1459	-0.082667	-0.10287	-1.1781	-1.4865	-1.69	0.2232	0.9836	1.0365
30	<0.01%	0.1161	0.27497	0.191488	-0.7807	0.806	0.1998	-0.3917	-0.2361	1.0365
11	<0.01%	-0.3295	0.305534	0.149801	-0.6591	-0.2266	-0.503	-0.9357	-0.2361	0.0915
26	<0.01%	-0.7371	0.211716	0.074298	-0.0232	-1.829	-1.3291	0.9376	-0.2361	1.0365
48	<0.01%	-0.9687	-0.287579	0.22685	0.248	-1.2356	-1.1487	-0.1869	-0.2361	1.0365
28	<0.01%	-1.344	0.094088	0.128227	-0.2664	-0.5503	-0.8638	0.5191	0.9836	-0.8536
17	<0.01%	-1.564	-4.938028	0.215849	-0.3786	-0.8203	-0.8163	1.2898	0.9836	0.0915

Supplementary Table 10. Multiple variable principal component analysis (from data in Supplementary Table 9)

Variable	PC1	PC2	PC3	PC4
Proliferation	0.11650344	-0.051460927	0.848595336	-0.152808964
in vitro FC	0.470123474	-0.100020373	0.576431359	0.244540003
In vivo FC	-0.254326176	0.220580657	-0.247651122	0.81824043
MHC-I	0.131873576	0.76046261	0.39387144	0.239461346
EpCAM	0.899044448	-0.248704834	-0.172348184	0.114295429
PD-L1	0.841540144	-0.012724457	-0.038112171	0.286703783
Zeb1	-0.689680784	0.325183209	0.344866696	0.111640342
CK8	0.11209978	0.793239222	-0.349805081	-0.228454557
CK14	-0.602447053	-0.628249934	0.034361493	0.269008536

Supplementary Table 11. Pearson correlation matrix of 7 biological features

	Proliferation	MHC-I	EpCAM	PD-L1	Zeb1	CK8	CK14
Proliferation	1.000	0.171	-0.005	0.021	0.136	-0.227	-0.080
MHC-I	0.171	1.000	-0.100	0.198	0.312	0.313	-0.416
EpCAM	-0.005	-0.100	1.000	0.870	-0.636	-0.087	-0.346
PD-L1	0.021	0.198	0.870	1.000	-0.373	0.030	-0.319
Zeb1	0.136	0.312	-0.636	-0.373	1.000	0.034	0.276
CK8	-0.227	0.313	-0.087	0.030	0.034	1.000	-0.611
CK14	-0.080	-0.416	-0.346	-0.319	0.276	-0.611	1.000
