# Supplementary material for: Clonal barcoding with qPCR detection enables live cell functional analyses for cancer research
Source: Nat Commun. 2022 Jul 4;13:3837. doi: 10.1038/s41467-022-31536-5 (PMC9252988; doi:10.1038/s41467-022-31536-5)
Supplement: Supplementary file 2 — Reporting Summary [file 41467_2022_31536_MOESM2_ESM.pdf]

nature portfolio

Corresponding author(s): Sandra McAllister

Last updated by author(s): May 24, 2022

Reporting Summary

Nature Portfolio wishes to improve the reproducibility of the work that we publish. This form provides structure for consistency and transparency in reporting. For further information on Nature Portfolio policies, see our Editorial Policies and the Editorial Policy Checklist.

Statistics

For all statistical analyses, confirm that the following items are present in the figure legend, table legend, main text, or Methods section.

n/a	Confirmed	
☐	☒	The exact sample size (n) for each experimental group/condition, given as a discrete number and unit of measurement
☐	☒	A statement on whether measurements were taken from distinct samples or whether the same sample was measured repeatedly
☐	☒	The statistical test(s) used AND whether they are one- or two-sided<br>*Only common tests should be described solely by name; describe more complex techniques in the Methods section.*
☐	☒	A description of all covariates tested
☒	☐	A description of any assumptions or corrections, such as tests of normality and adjustment for multiple comparisons
☐	☒	A full description of the statistical parameters including central tendency (e.g. means) or other basic estimates (e.g. regression coefficient) AND variation (e.g. standard deviation) or associated estimates of uncertainty (e.g. confidence intervals)
☐	☒	For null hypothesis testing, the test statistic (e.g. F, t, r) with confidence intervals, effect sizes, degrees of freedom and P value noted<br>*Give P values as exact values whenever suitable.*
☒	☐	For Bayesian analysis, information on the choice of priors and Markov chain Monte Carlo settings
☐	☒	For hierarchical and complex designs, identification of the appropriate level for tests and full reporting of outcomes
☐	☒	Estimates of effect sizes (e.g. Cohen's d, Pearson's r), indicating how they were calculated

Our web collection on statistics for biologists contains articles on many of the points above.

Software and code

Policy information about availability of computer code

Data collection	qPCR was performed using Appliled Biosystems 7900HT. Images were captured with Nikon NIS Elements v4.3. Flow cytometric acquisition was performed with BD FACSDiva software v9.0 (BD Biosciences). Bioluminescence was captured using IVIS Lumina III imaging system and Living Image v4.1 (Caliper Life Sciences)
Data analysis	Prism v9.3.1, NIH ImageJ (http://imagej.nih.gov/ij/), FlowJo v10.5.3, Living Image v4.1 (Caliper Life Sciences). Custom codes developed for the study can be found at: https://github.com/petervangalen/BarcodeSimilarity or https://doi.org/10.5281/zenodo.6585760

For manuscripts utilizing custom algorithms or software that are central to the research but not yet described in published literature, software must be made available to editors and reviewers. We strongly encourage code deposition in a community repository (e.g. GitHub). See the Nature Portfolio guidelines for submitting code & software for further information.

Data

Policy information about availability of data

All manuscripts must include a data availability statement. This statement should provide the following information, where applicable:

- Accession codes, unique identifiers, or web links for publicly available datasets
- A description of any restrictions on data availability
- For clinical datasets or third party data, please ensure that the statement adheres to our policy

Source data are provided as a Source Data file. The following datasheets can be found online: Supplementary Table 1: Information on collection of clonally barcoded cell lines; Supplementary Table 2. Oligonucleotide primer sequences for qPCR-based BC detection; Supplementary Table 3. Barcode-index reverse (R) primer sequences for NGS-based BC detection; Supplementary Table 4. Illumina library preparation primer sets; Supplementary Table 5. Composition of Met1 BC pool at start and end of injections; Supplementary Table 6. Raw data for biological features of individual Met1 BCs; Supplementary Table 7. Z scores for biological features

of individual Met1 BCs; Supplementary Table 8. Pearson correlation matrix from measurements of Met1 BC biological features; Supplementary Table 9. Multiple variable Z scores used for principal component analysis; Supplementary Table 10. Multiple variable principal component analysis; Supplementary Table 11. Pearson correlation matrix of 7 biological features assessed for Met1 BCs. Next generation sequencing raw data for barcode identification are available upon request

Field-specific reporting

Please select the one below that is the best fit for your research. If you are not sure, read the appropriate sections before making your selection.

[x] Life sciences [ ] Behavioural & social sciences [ ] Ecological, evolutionary & environmental sciences

For a reference copy of the document with all sections, see nature.com/documents/nr-reporting-summary-flat.pdf

Life sciences study design

All studies must disclose on these points even when the disclosure is negative.

Sample size	Sample sizes were chosen based on previous experience with the models and methods used in this study (see doi: 10.1158/2159-8290.CD-18-1454)
Data exclusions	Tumors that were found to have grown subcutaneously rather than in the mammary fat pat were excluded from analysis. Those cases are clearly noted in the Data Source file included with this manuscript
Replication	Biological experiments were repeated at least twice whenever possible and all attempts at replication were successful.
Randomization	Animals were randomly assigned to groups. Otherwise, sample allocation for cell culture experiments was not applicable to this study.
Blinding	Investigators were not blinded to allocation during initiation of experiments, as accuracy and appropriate cell injections into mice was critical to the studies. Following injection, investigators where then blinded to animal/cage identification. Downstream analyses (tumor measurements, metastasis quantification, immunofluorescence, image analysis, flow cytometry) were performed in a blinded fashion.

Reporting for specific materials, systems and methods

We require information from authors about some types of materials, experimental systems and methods used in many studies. Here, indicate whether each material, system or method listed is relevant to your study. If you are not sure if a list item applies to your research, read the appropriate section before selecting a response.

Materials & experimental systems

n/a	Involved in the study	
[ ]	[x]	Antibodies
[ ]	[x]	Eukaryotic cell lines
[x]	[ ]	Palaeontology and archaeology
[ ]	[x]	Animals and other organisms
[x]	[ ]	Human research participants
[x]	[ ]	Clinical data
[x]	[ ]	Dual use research of concern

Methods

n/a	Involved in the study	
[x]	[ ]	ChIP-seq
[ ]	[x]	Flow cytometry
[x]	[ ]	MRI-based neuroimaging

Antibodies

Antibodies used	For Immunofluorescence:<br>F-actin (rhodamine phalloidin, 1:1000; Thermo Fisher catalog: R415)<br>CK14 (1:200; Biolegend catalog: 905303)<br>CK8 (1:10; TROMA-1, DSHB).<br>anti-Zeb1 (1:100; Santa Cruz Biotechnology catalog: sc-25388)<br>Secondary Alexa Fluor antibodies (Invitrogen, 1:250): anti-rat 647 (catalog: A21472), anti-rabbit 488 (catalog: A21206), anti-rat 488 (catalog: A21208), anti-rabbit 647 (catalog: A21244).<br><br>For Flow cytometry:<br>anti-EpCAM (Clone G8.8, APC-Cy7, 1:400 dilution, BioLegend: 118218)<br>anti-MHC-I (clone: KH114, FITC, 1:400 dilution, BioLegend: 115104)<br>PD-L1 (clone: 10F.9G2, PE, 1:100 dilution, BioLegend: 124308)<br>anti-CD16/32 FcУ III/II receptor antibody (BioLegend) used at a concentration of 250 ng/10^6 cells
Validation	For flow cytometry and immunofluorescence, antibodies were validated as noted on manufacturer's website, and most of antibodies specificity was confirmed in the literature. In addition, the stainings were consistent with the predicted cellular localization of the protein.

CK8 https://dshb.biology.uiowa.edu/TROMA-I
CK14 https://www.biolegend.com/fr-fr/products/purified-anti-keratin-14-antibody-13317
Zeb1 https://www.scbt.com/p/zeb1-antibody-h-102
Secondary antibodies https://www.thermofisher.com/us/en/home/brands/molecular-probes/key-molecular-probes-products/alexa-fluor/alexa-fluor-products.html
EpCAM https://www.biolegend.com/fr-ch/products/apc-cyanine7-anti-mouse-cd326-ep-cam-antibody-5577
MHC-I https://www.biolegend.com/en-us/products/fitc-anti-mouse-h-2kq-antibody-1585
PD-L1 https://www.biolegend.com/it-it/products/pe-cyanine7-anti-mouse-cd274-b7-h1-pd-l1-antibody-6721

Eukaryotic cell lines

Policy information about cell lines

Cell line source(s)	The Met1 TNBC cell line, which was originally generated from a spontaneously arising tumor in an FVB/N-Tg (MMTV-PyVmT) mouse, was a gift from J. Joyce (University of Lausanne) with permission from A. Borowsky. The 4T1 TNBC cell line, which was derived from a spontaneously arising tumor in a Balb/c mouse, was originally generated and provided by F. Miller (Wayne State University School of Medicine). The McNeuA HER2+ cell line, which was originally derived from a spontaneously arising breast tumor in an MMTV-neu transgenic mouse, was provided by Michael Campbell (University of California, San Francisco). Human HMLER-hygro-H-rasV12 (HMLER-HR) cells were a gift from R. Weinberg (derived from human mammary epithelial cells originally obtained from ATCC). 293T cells were purchased directly from ATCC.
Authentication	All cell lines were authenticated by short tandem repeat analysis.
Mycoplasma contamination	All cell lines were routinely tested to confirm the absence of mycoplasma contamination.
Commonly misidentified lines (See ICLAC register)	No commonly misidentified cell lines were used.

Animals and other organisms

Policy information about studies involving animals; ARRIVE guidelines recommended for reporting animal research

Laboratory animals	Female FVB mice and BALB/cJ mice 8-9 weeks of age were purchased from Jackson Laboratory.
Wild animals	No wild animals were used in the study.
Field-collected samples	No field-collected samples were used in the study.
Ethics oversight	All animal studies were conducted in accordance with regulations of the Institutional Animal Care and Use Committee of the Brigham and Women’s Hospital (protocol no. 2017N000056).

Note that full information on the approval of the study protocol must also be provided in the manuscript.

Flow Cytometry

Plots

Confirm that:

- [x] The axis labels state the marker and fluorochrome used (e.g. CD4-FITC).
- [x] The axis scales are clearly visible. Include numbers along axes only for bottom left plot of group (a 'group' is an analysis of identical markers).
- [x] All plots are contour plots with outliers or pseudocolor plots.
- [x] A numerical value for number of cells or percentage (with statistics) is provided.

Methodology

Sample preparation	Cell suspensions were blocked with anti-CD16/32 FcУ III/II receptor antibody (BioLegend) used at a concentration of 250 ng/10^6 cells for 30 minutes on ice. After washing, cells were incubated with anti-EpCAM-APC-Cy7 (Clone: G8.8, 1:400 dilution), anti-MHC-I-FITC (clone: KH114, 1:400 dilution), anti-PD-L1-PE-Cy7 (clone: 10.F.9G2, 1:100 dilution). DAPI (0.1 µg/ml, Sigma) was used as a dead cell marker.
Instrument	Samples were analyzed on a BD Canto II.
Software	FACSDiva Software (BD Biosciences).
Cell population abundance	All flow cytometry was performed on cultured cells for this study.
Gating strategy	For all experiments, debris was first excluded by a morphology gate based on FSC-A and SSC-A. Then, non-singlets were

eliminated from analysis by a single cell gate based on FSC-H and FSC-A. Next, dead cells were eliminated by an appropriate viability gate: DAPI was used to distinguish live/dead cells.

☒ Tick this box to confirm that a figure exemplifying the gating strategy is provided in the Supplementary Information.
